# Supplementary material for: Glucose and Cell Context-Dependent Impact of BMI-1 Inhibitor PTC-209 on AKT Pathway in Endometrial Cancer Cells
Source: Cancers (Basel). 2022 Dec 1;14(23):5947. doi: 10.3390/cancers14235947 (PMC9739103; doi:10.3390/cancers14235947)
Supplement: Supplementary file 1 [file cancers-14-05947-s001.zip › cancers-1973946-Tabs and Figs.pdf]

Supplementary Materials

Glucose and Cell Context-Dependent Impact of BMI-1 Inhibitor PTC-209 on AKT Pathway in Endometrial Cancer Cells

Table S1. The identification number of TaqMan® probes or primers sequence used in the study.

Gene	TaqMan® probes/ primers sequence
PP2A	Hs00603515_m1
PHLPP1	Hs01597871_m1
PHLPP2	Hs00982295_m1
INPP4B	Hs01038089_m1
INPP5D	Hs00183290_m1
HPRT1	Hs02800695_m1
BMI1	Hs00180411_m1
BMI1	F: AATTAGTTCCAGGGCTTTTCAA<br>R: CTTCATCTGCAACCTCTCCTCTAT
PTEN	F: ACAGCCATCATCAAAGAGATCGT<br>R:TGCTTTGAATCCAAAAACCTTACTA
PHLPP1	F: AAACCTCACAGCACGGGTAG<br>R: AGGCAGGTCCCACATAGGAT
PHLPP2	F: TCCTGACCTCGGCTGTATGA<br>R: GGGTCTTTCCCTTGCGTACA
SNAIL	F: TGTCAACAGTACCACTGCCA<br>R: CCGGACTCTTGGTGCTTGTG
SLUG	F: AGAGCATTTGCAGACAGGTCA<br>R: CTACACAGCAGCCAGATTCCT
ZEB1	F: AAAGATGATGAATGCGAGTC<br>R: TCCATTTTCATCATGACCAC
TWIST	F: CTAGATGTCATTGTTTCCAGAG<br>R: CCCTGTTTCTTTGAATTTGG
CDH1	F: TACATCTCCCTTCACAGC<br>R: ATAGATTCTTGGGTTGGGTC

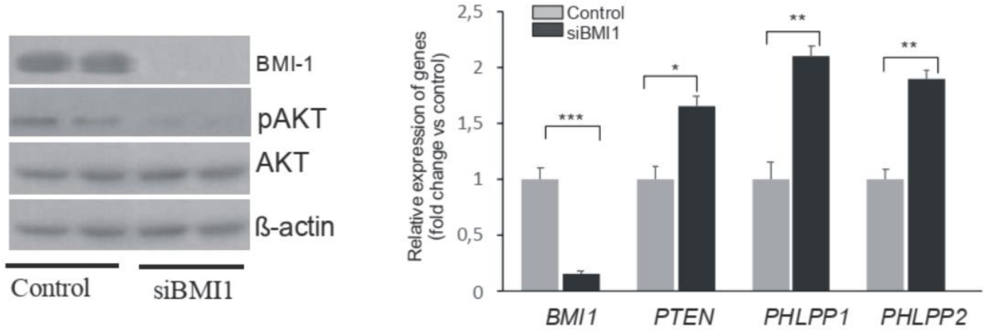

Figure S1. Effect of BMI1 expression silencing on AKT phosphorylation and gene expression in Ishikawa cells. Knockdown experiments were performed using Silencer Select siRNA (ID: S2016) (Ambion®, Carlsbad, CA USA). In controls, cells were treated with Silencer Select siRNA negative control. To knockdown of BMI-1 30 nmol/l siRNA and Lipofectamine RNAiMAX (Invitrogen, ThermoFisher Scientific, Grand Island, NY, USA) were used following manufacturer's specifications. The effect of BMI-1 silencing was checked after 48h. The figure shows the means +/-SD for three experiments performed in triplicate. * *p*. values <0.05 values; ** *p* values of <0.01; *** *p* values <0.001.

Table S2. The results of analysis of the correlation between BMI-1, PTEN and PHLPPs in cells treated with 0.5, 1, and 5 mM of PTC-209.

	HEC-1A		ISHIKAWA	
	0.5 mM glucose	30 mM glucose	0.5 mM glucose	30 mM glucose
BMI-1/PTEN	**r=-0,7870 p=0,0279**	r=-0.216 p=0.619	r=-0.708 p=0.291	r=-0.910 p=0.089
BMI-1/PHLPP1	r=0.870 p=0.279	r= -0.720 p= 0.057	r=0.856 p=0.442	**r= -0.970 p=0.029**
BMI-1/PHLPP2	r=0.097 p=0.840	**r=-0.926 p=0.046**	r= 0.305 p=0.695	**r=-0,985 p=0.015**

The correlation between BMI-1 protein and *PTEN*, *PHLPP1,* and *PHLPP2* mRNA expressions in cells treated with different PTC-209 concentrations was analyzed. The protein level was assessed by densitometric analysis and mRNA by RT-PCR. The experiment was performed in triplicate. Spearman correlation coefficient was calculated for correlation analysis. A p-value < 0.05 was considered to indicate a statistically significant difference. The results showed inverse correlation between BMI-1 expression and PHLPPs expression but only in hyperglycemia condition.
