# Supplementary figures and images for: Glucose and Cell Context-Dependent Impact of BMI-1 Inhibitor PTC-209 on AKT Pathway in Endometrial Cancer Cells
Source: Cancers (Basel). 2022 Dec 1;14(23):5947. doi: 10.3390/cancers14235947 (PMC9739103; doi:10.3390/cancers14235947)

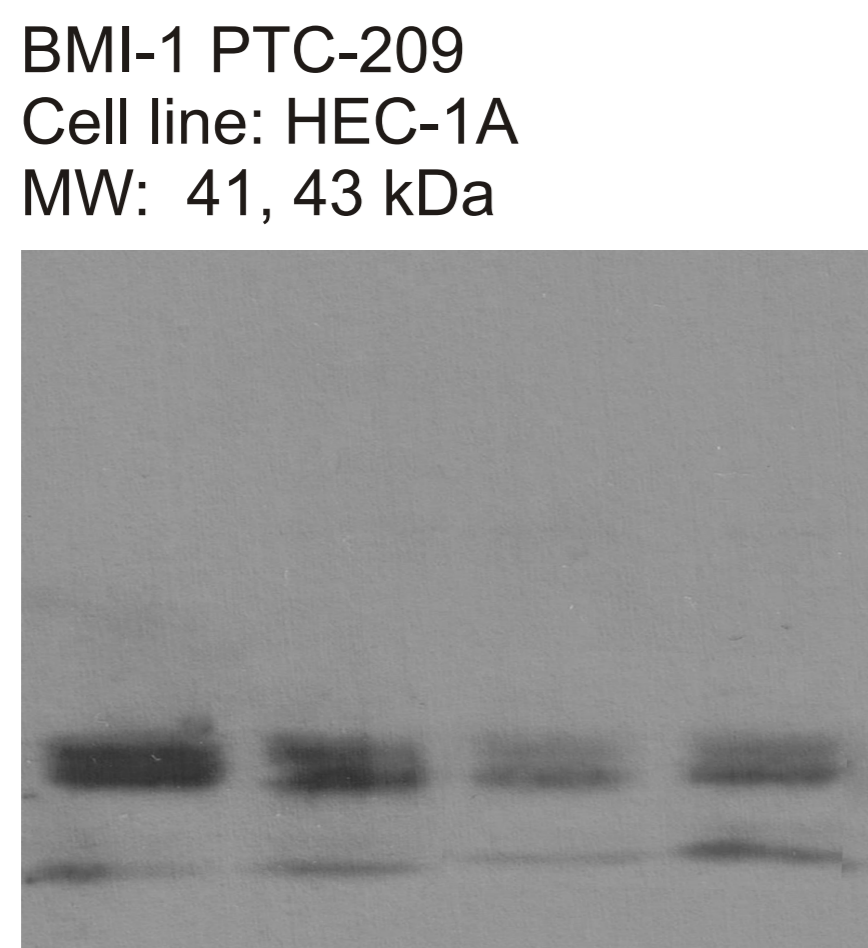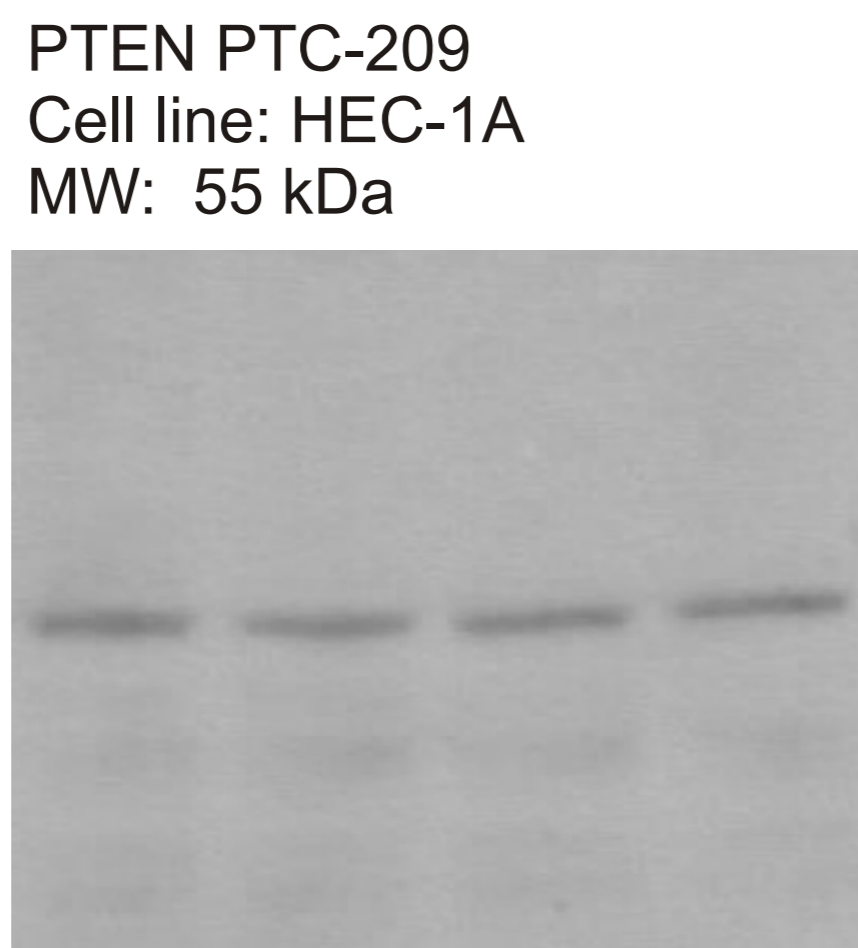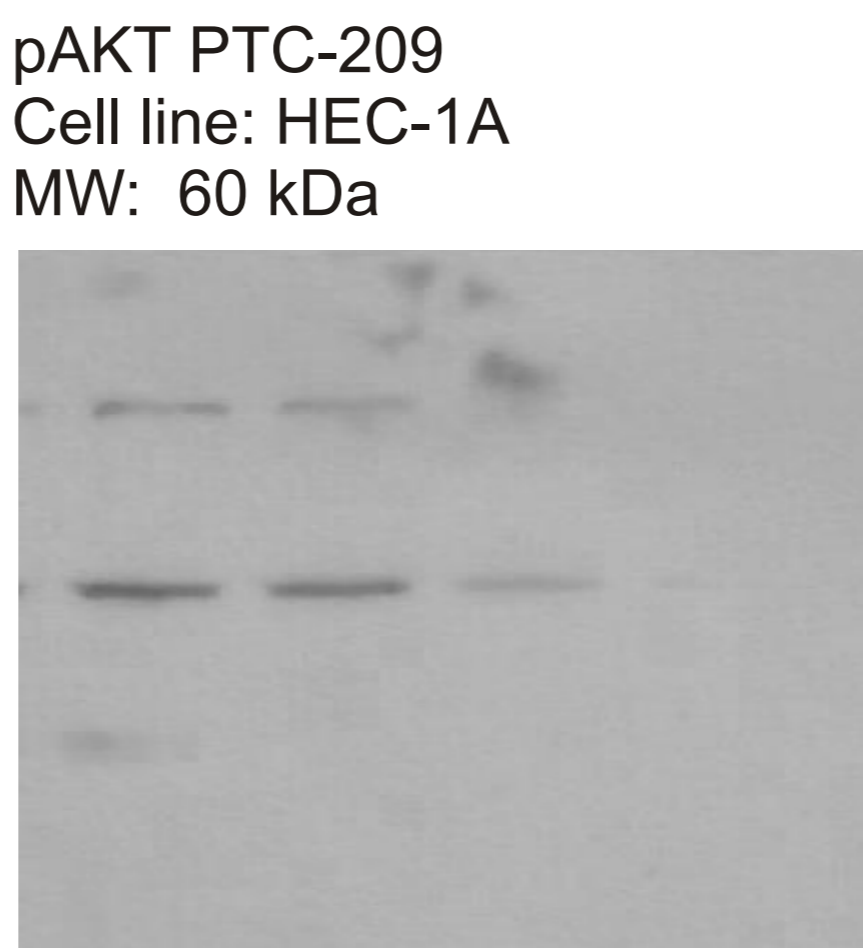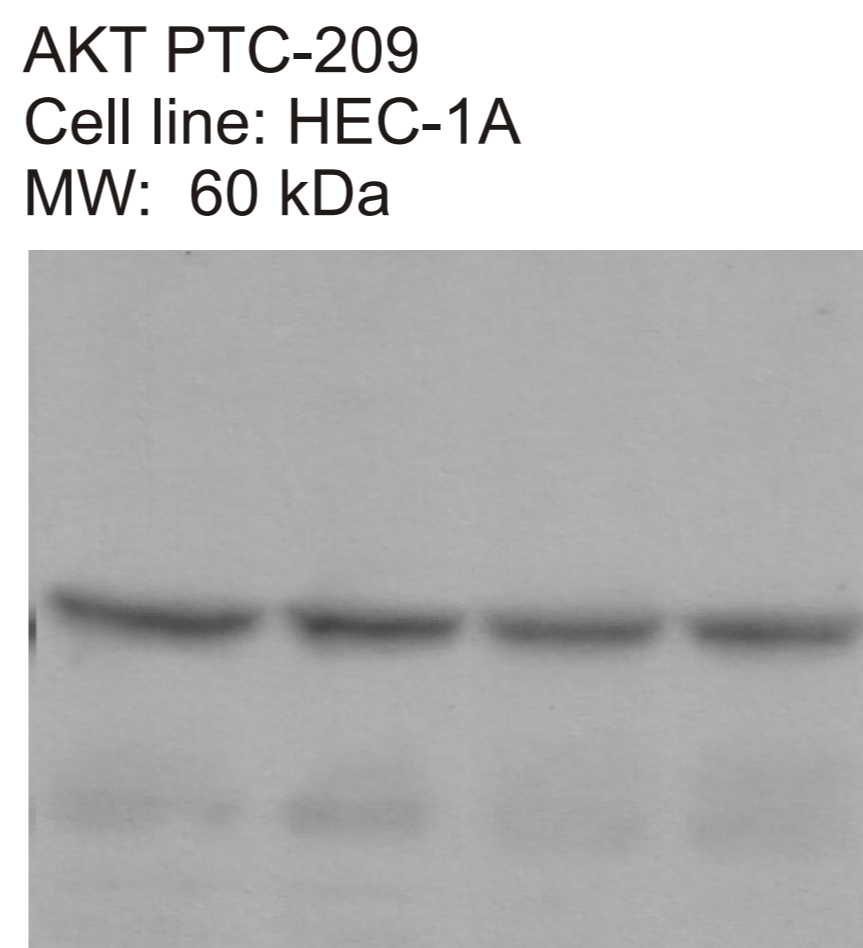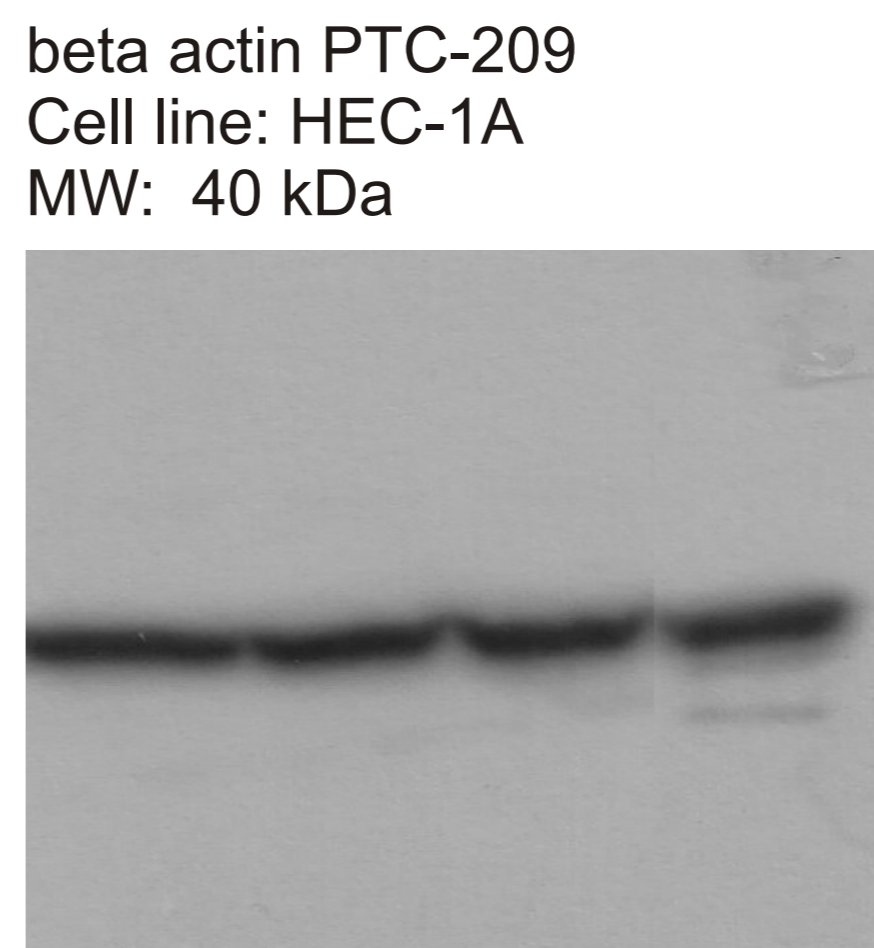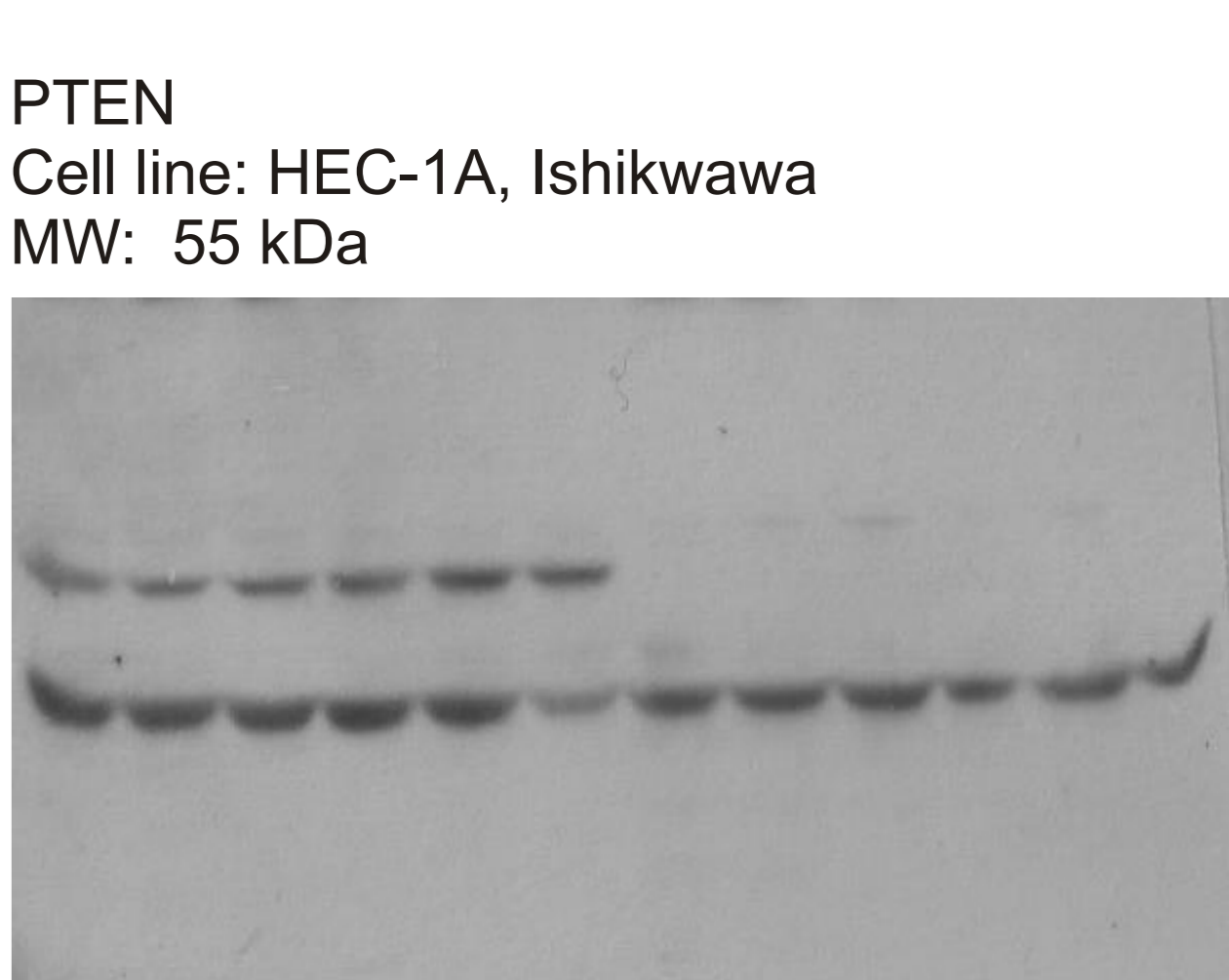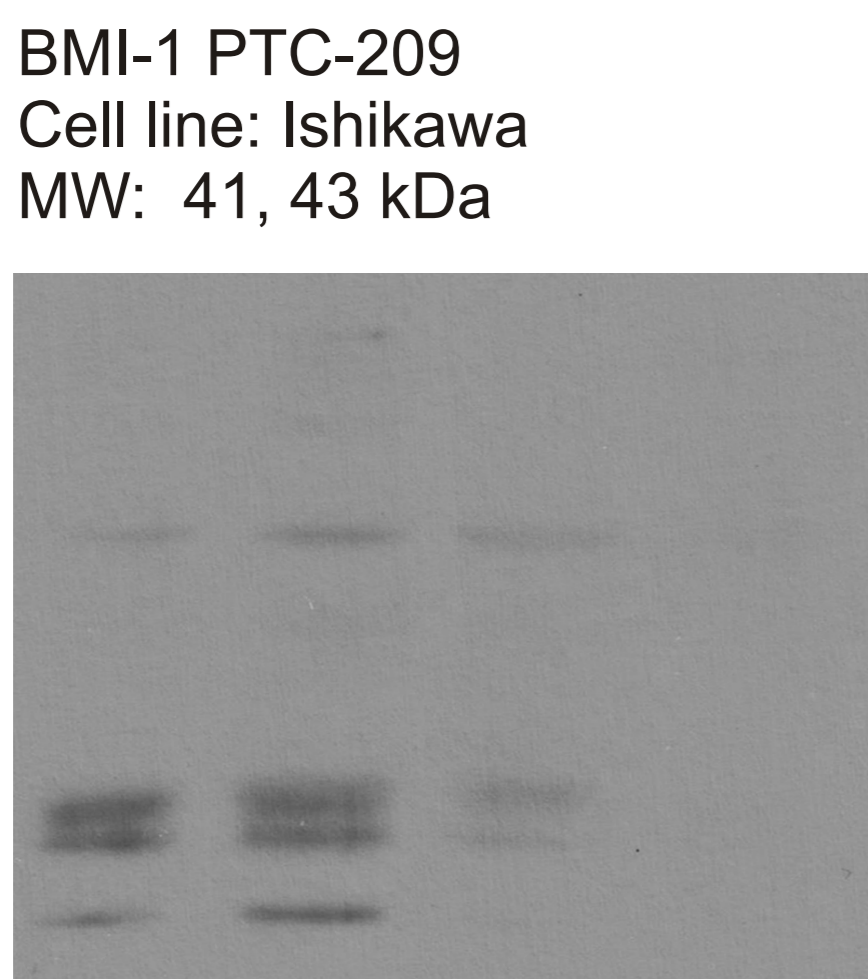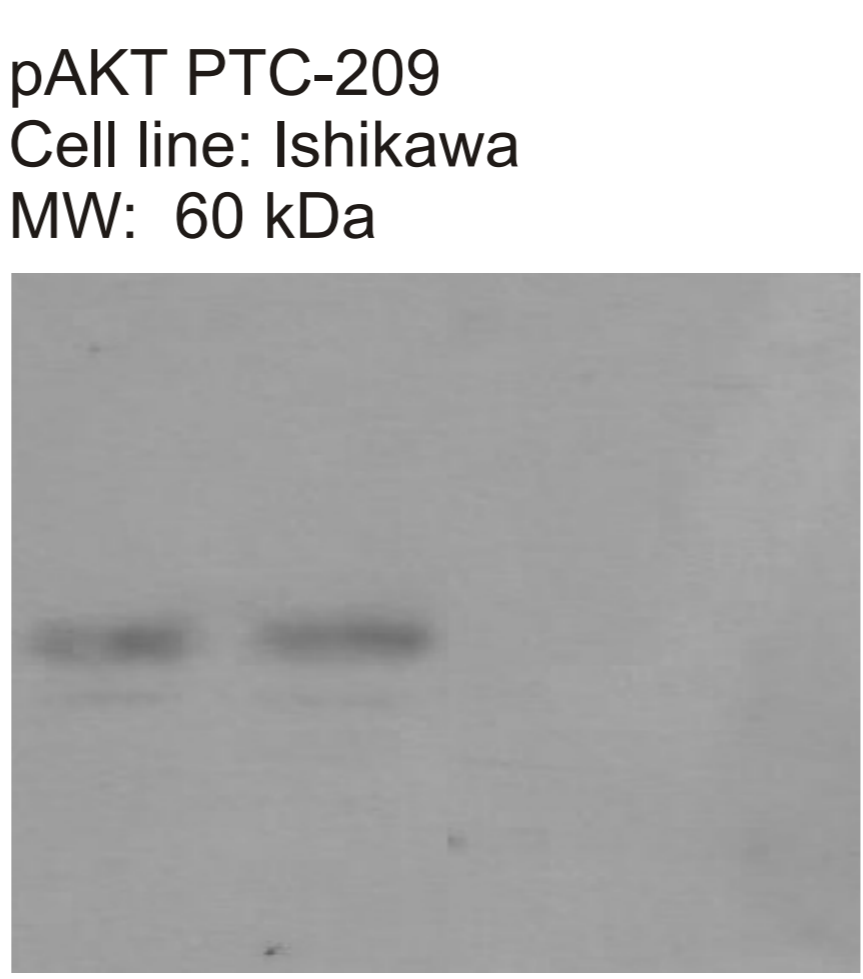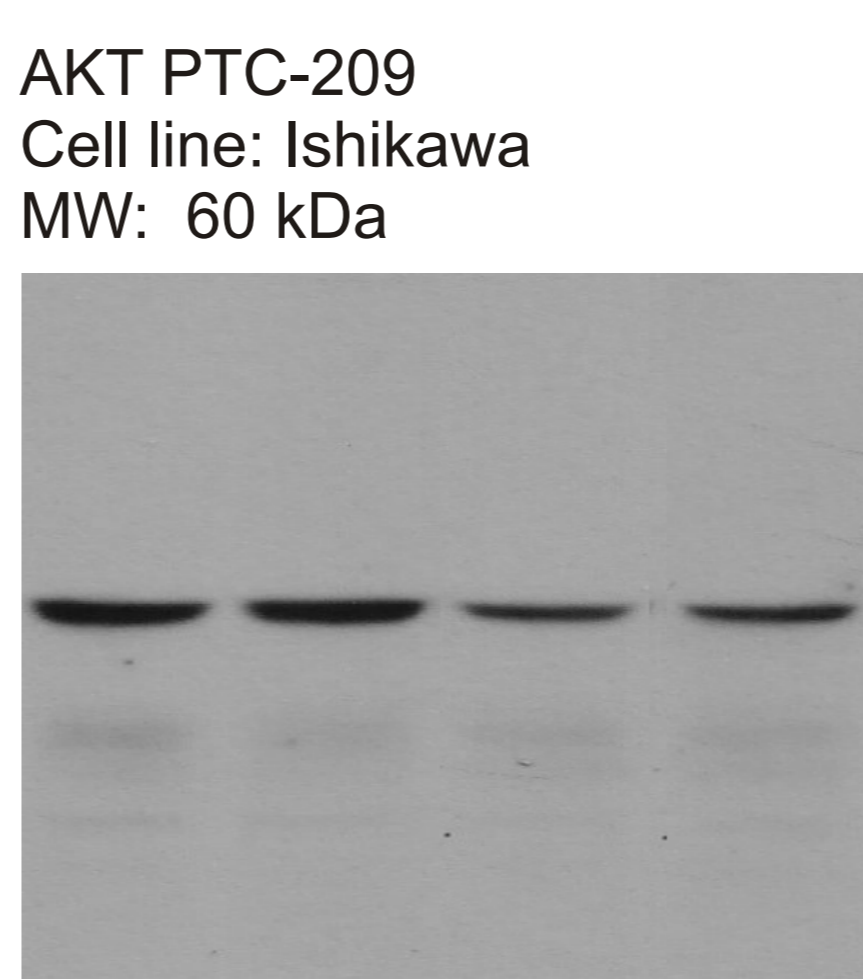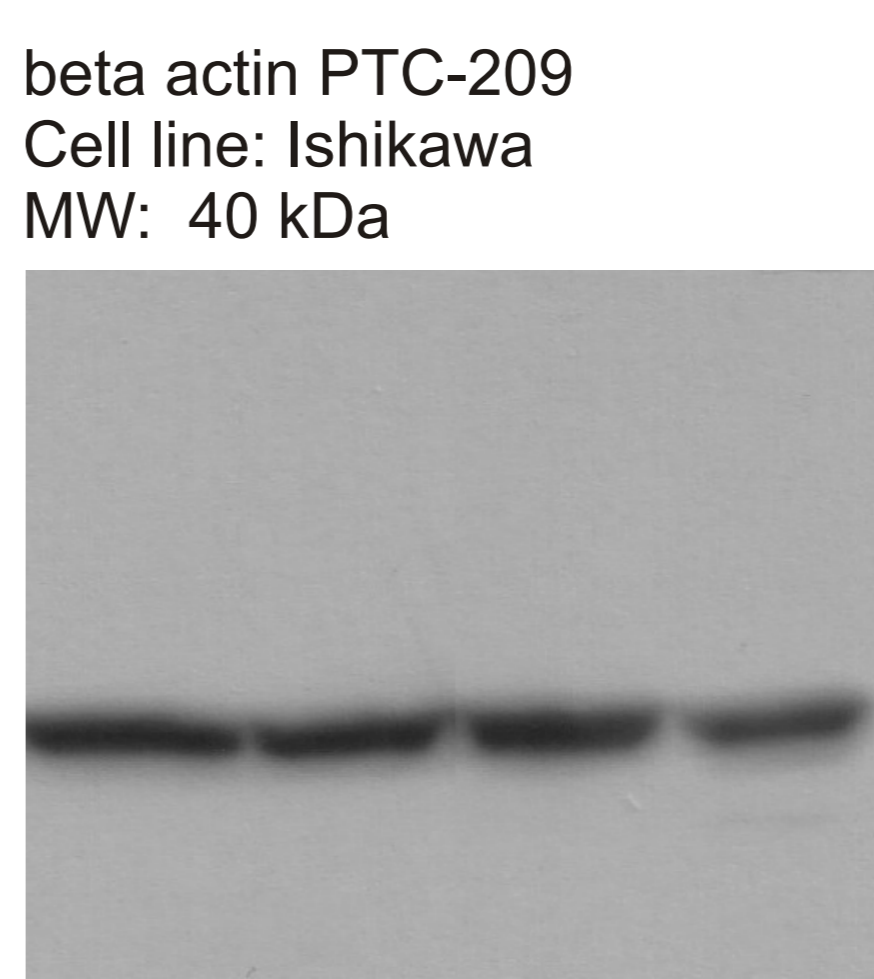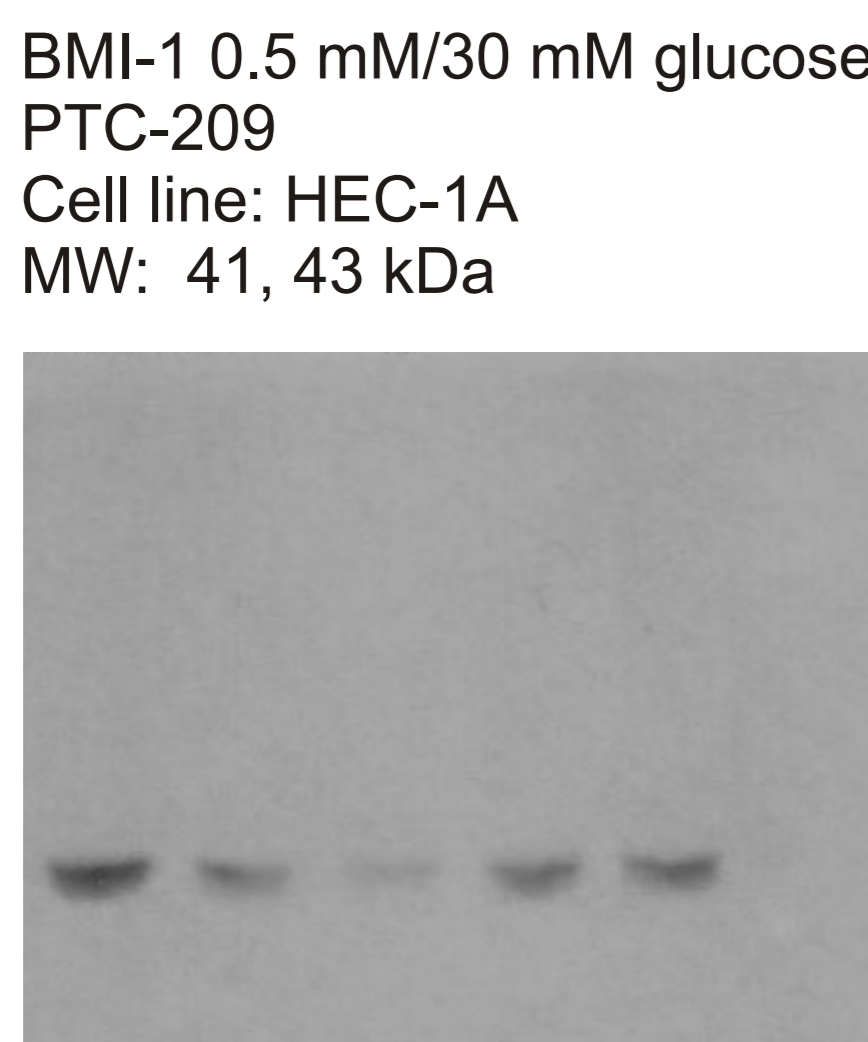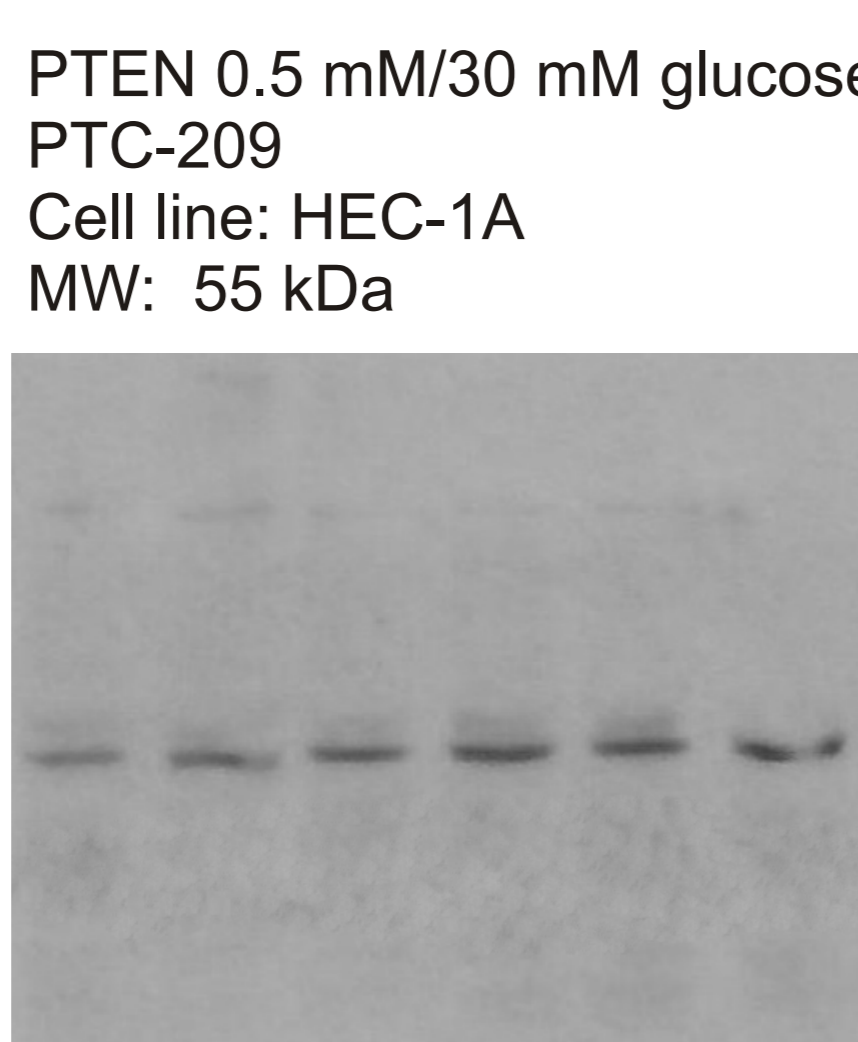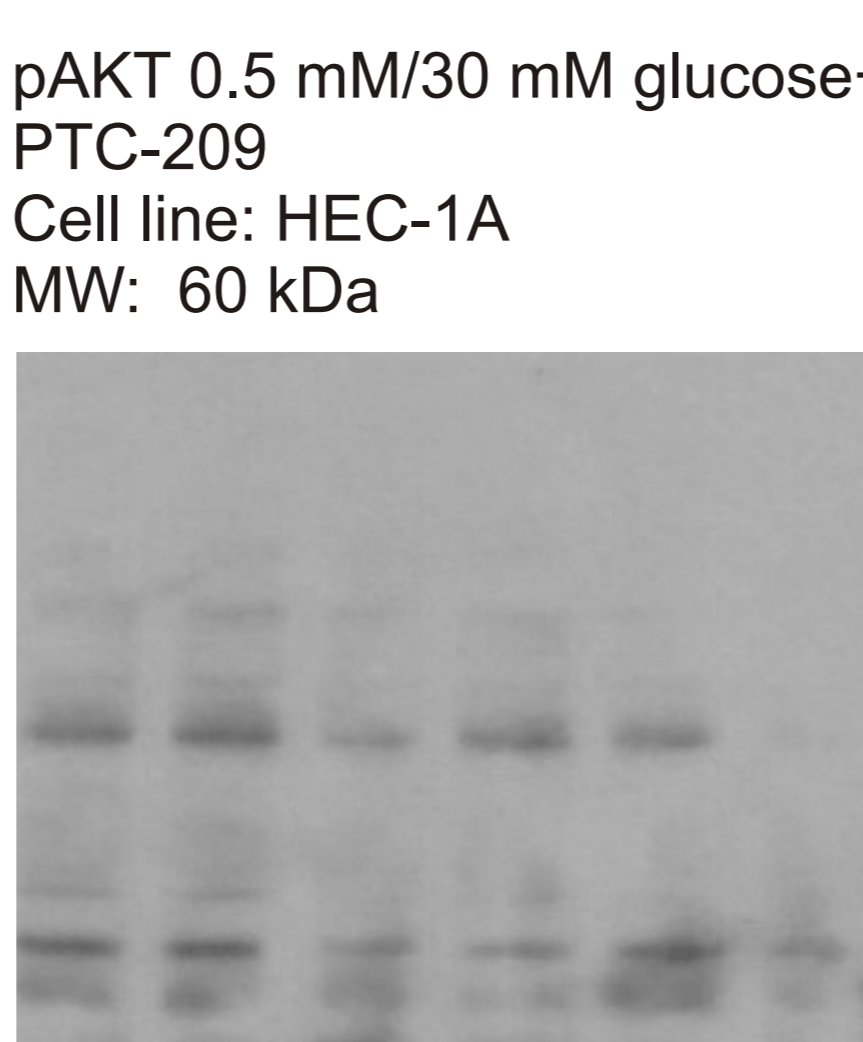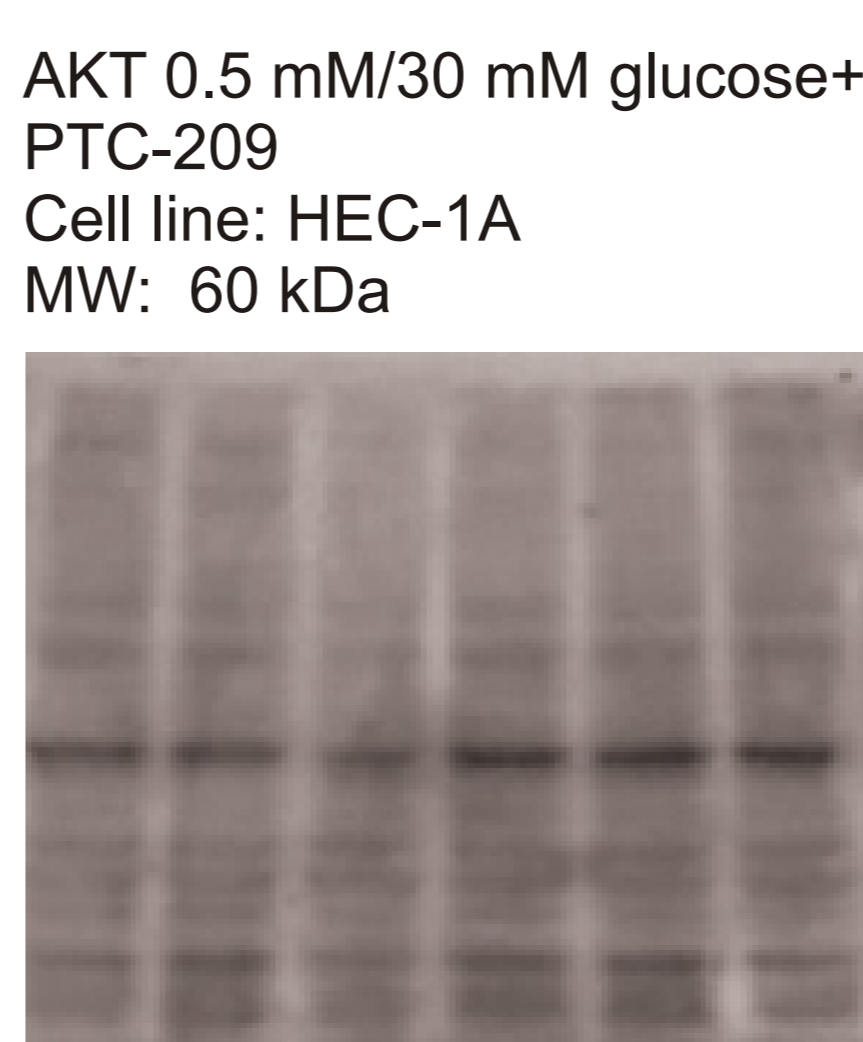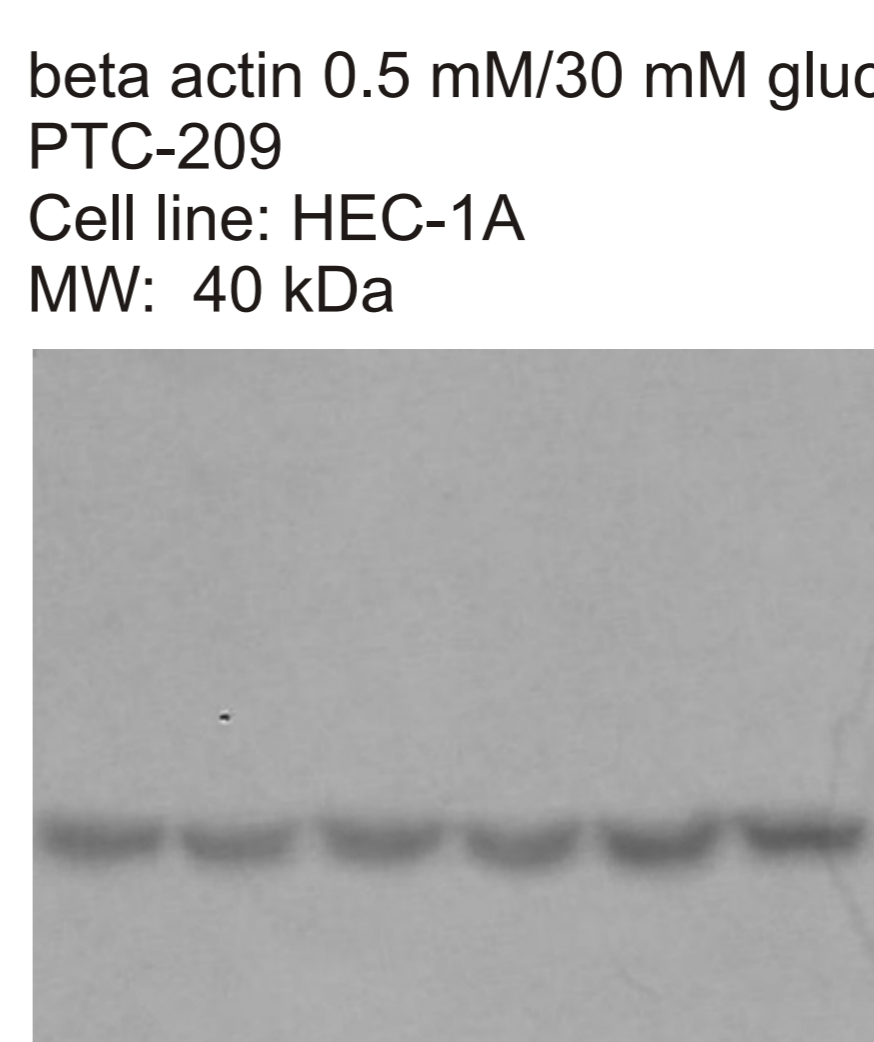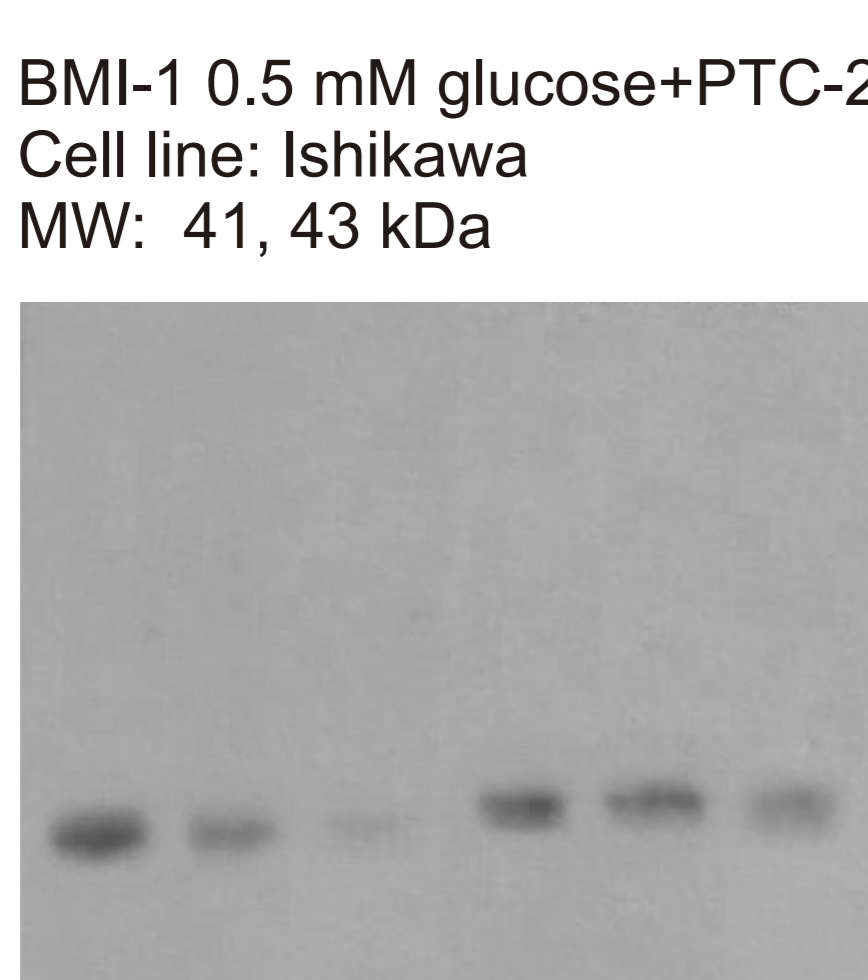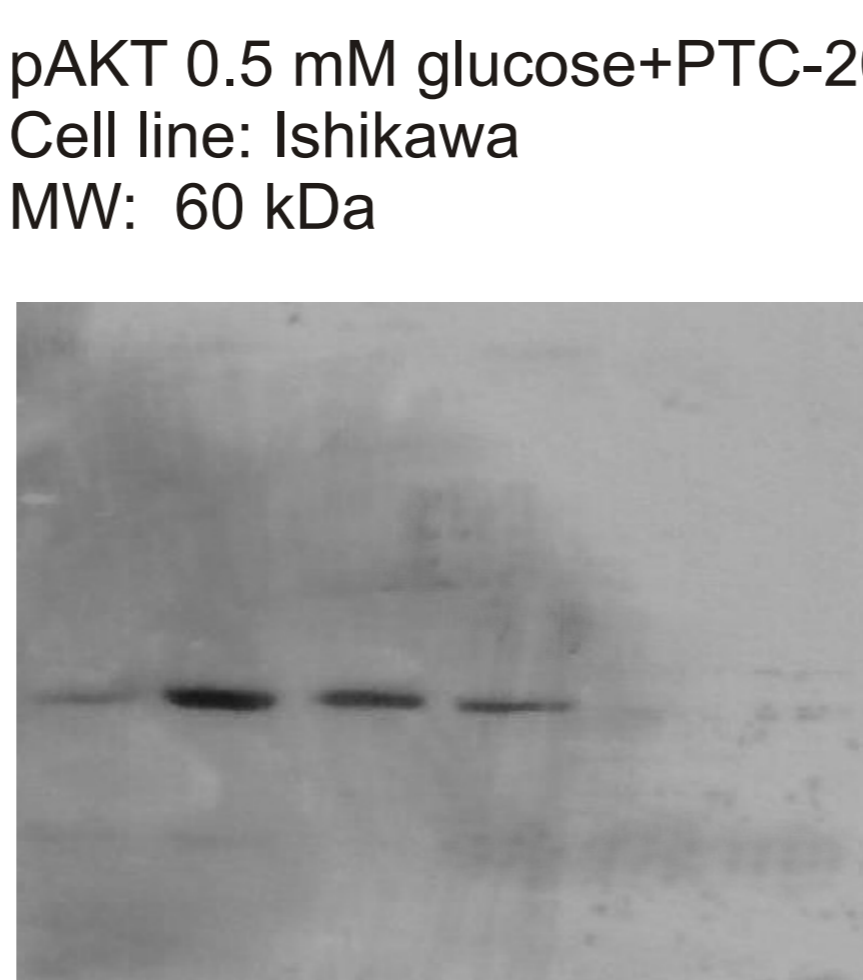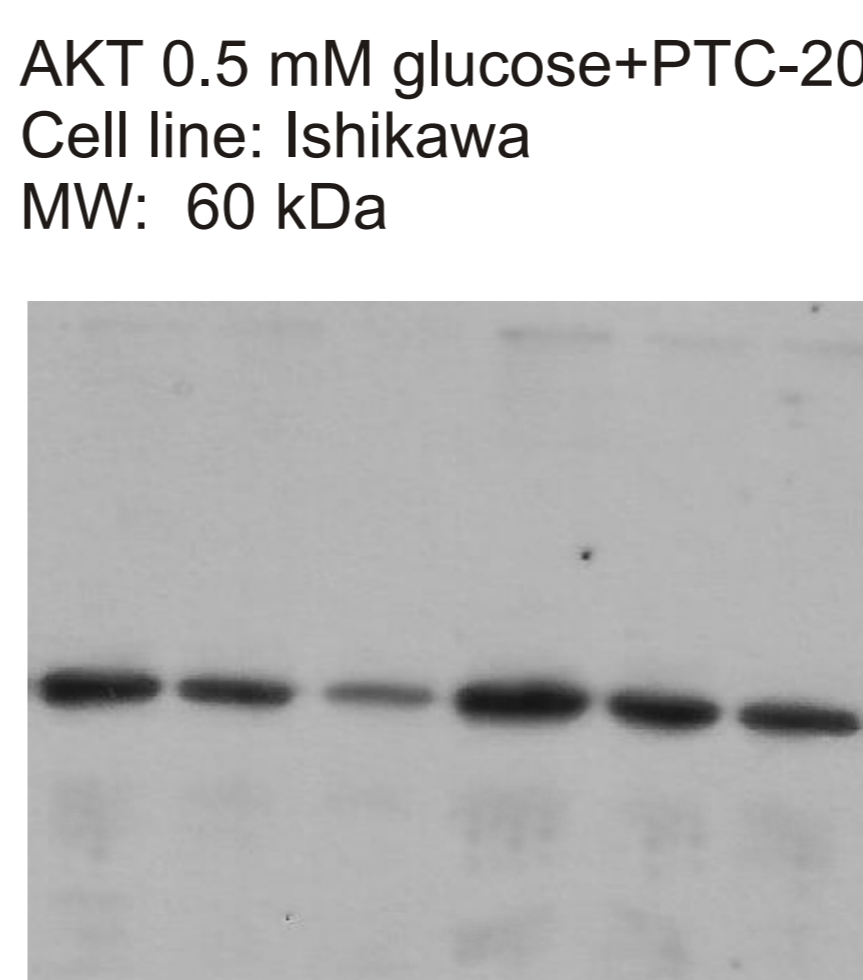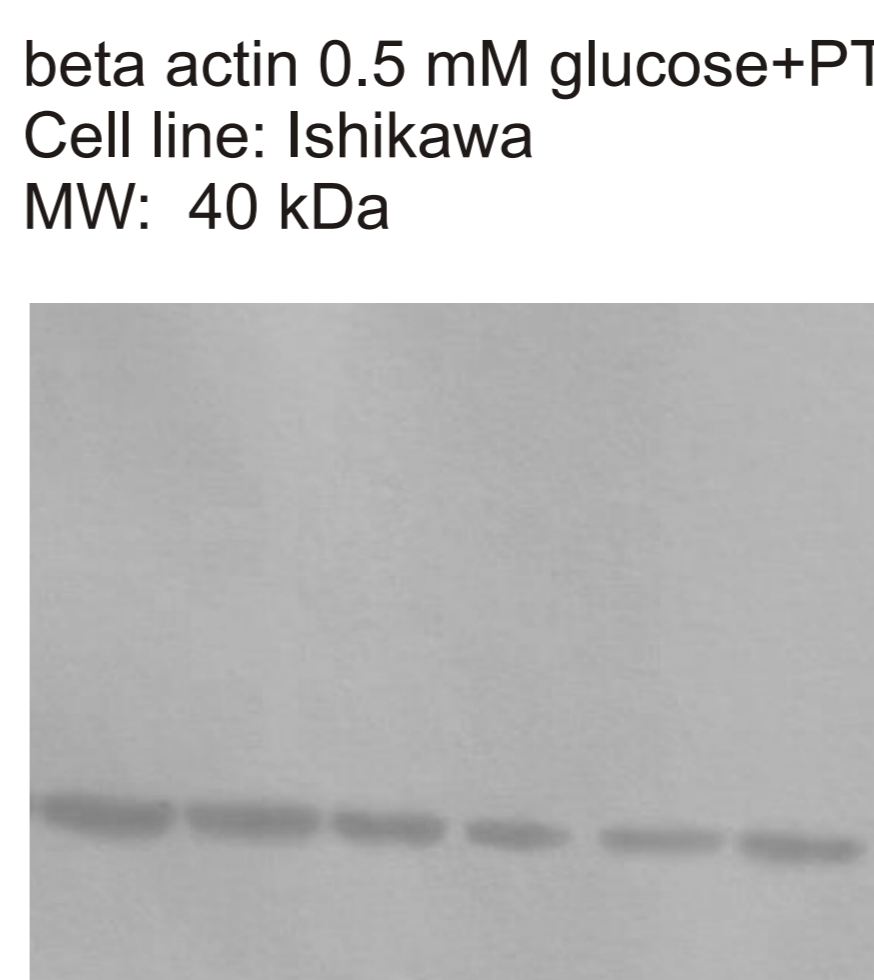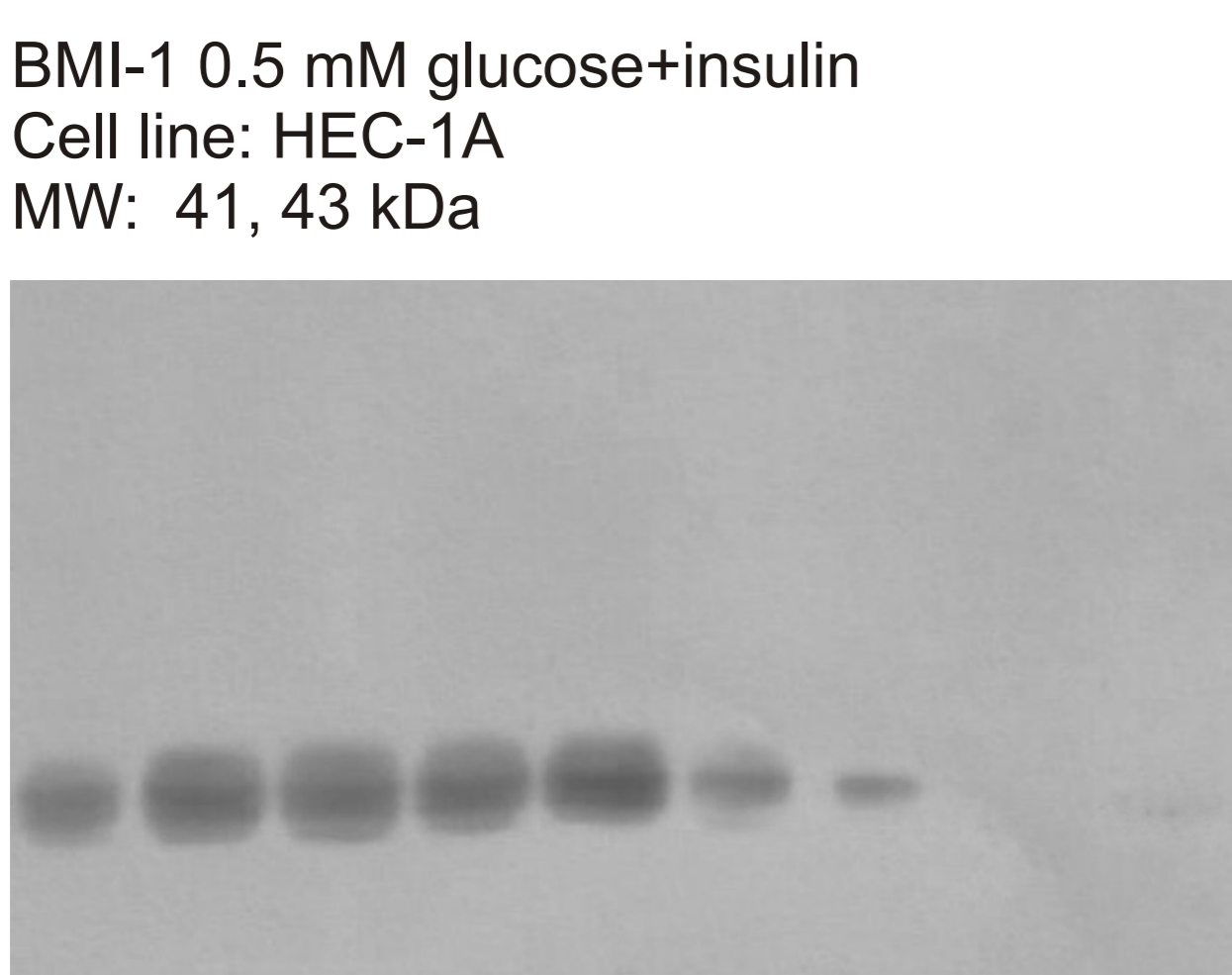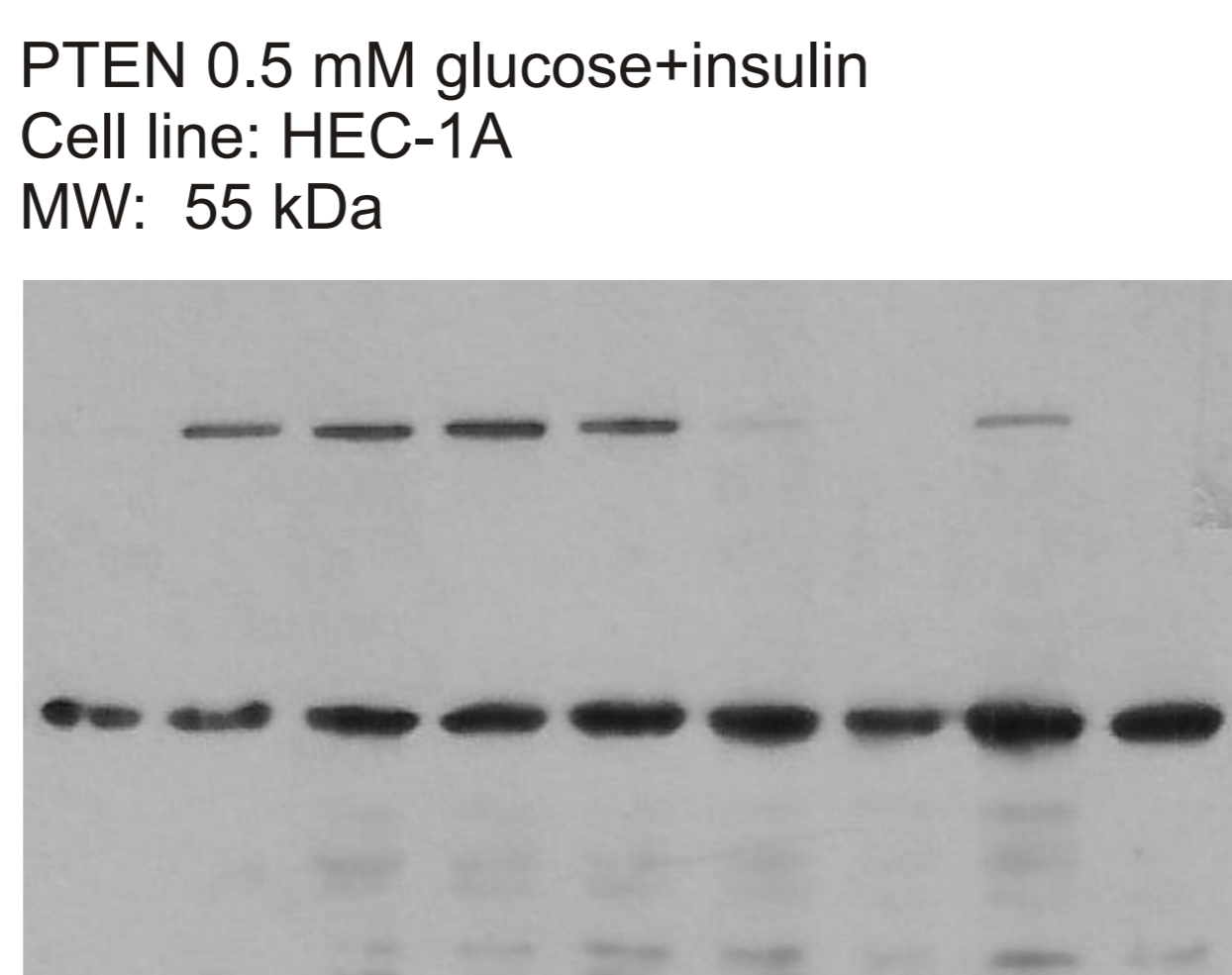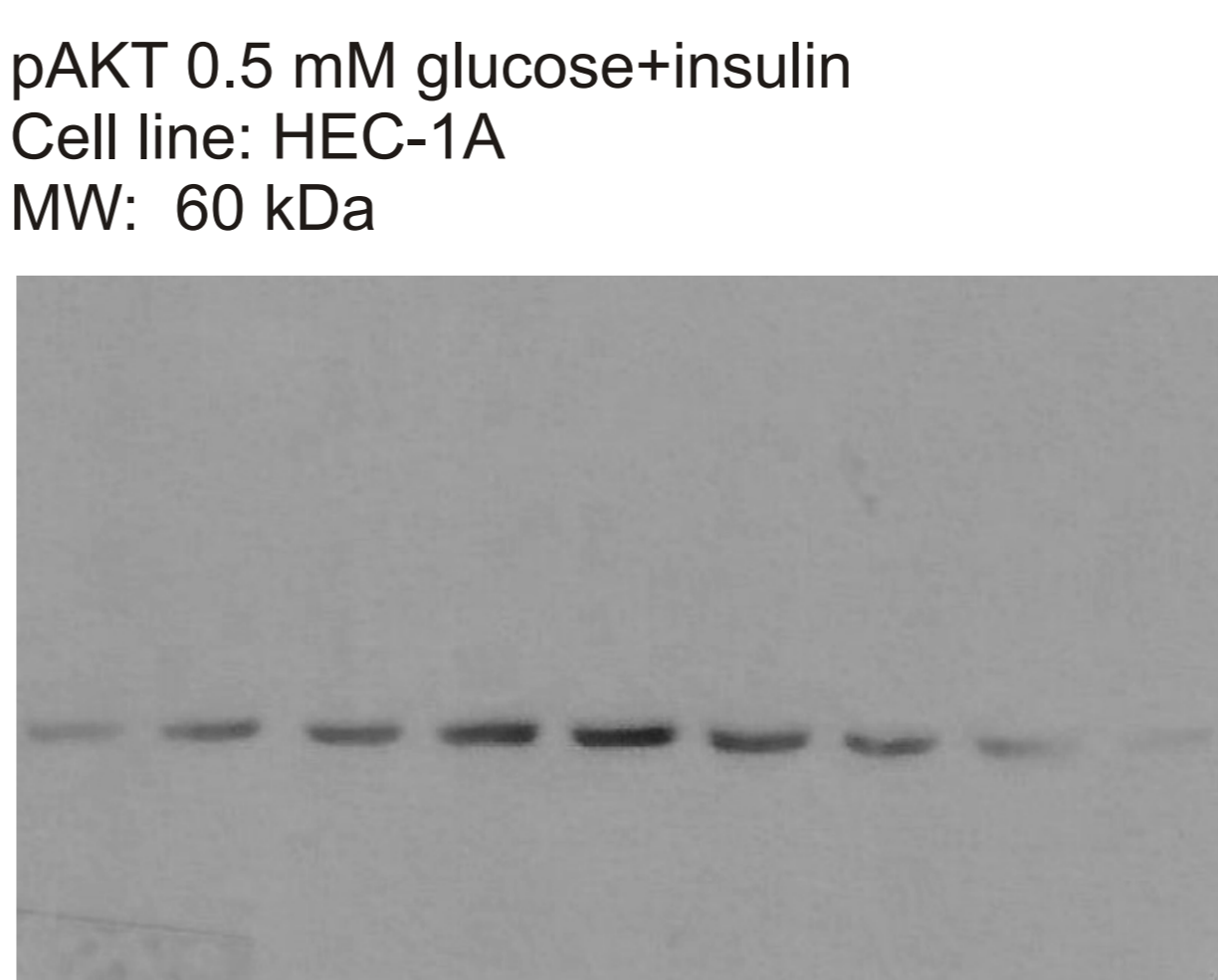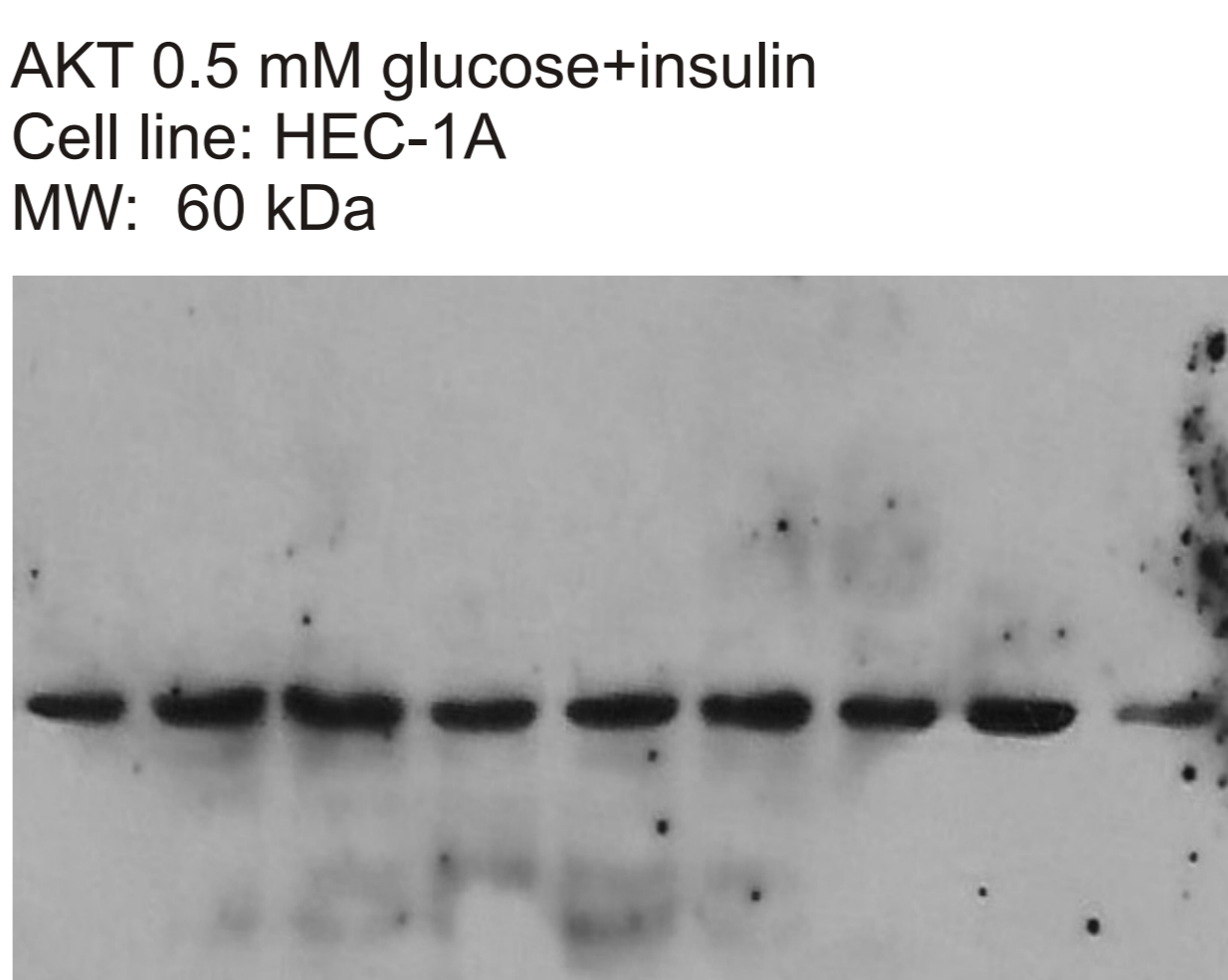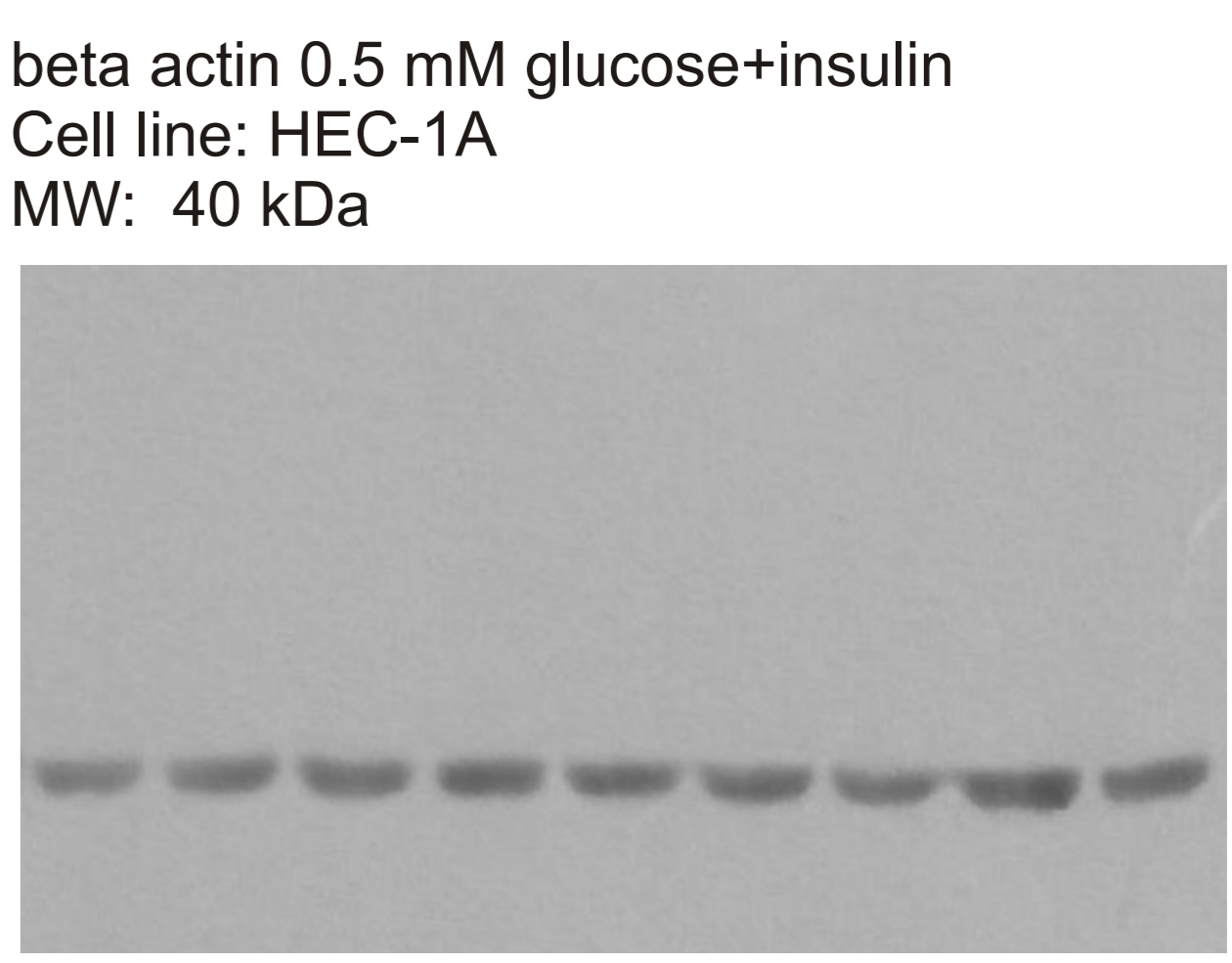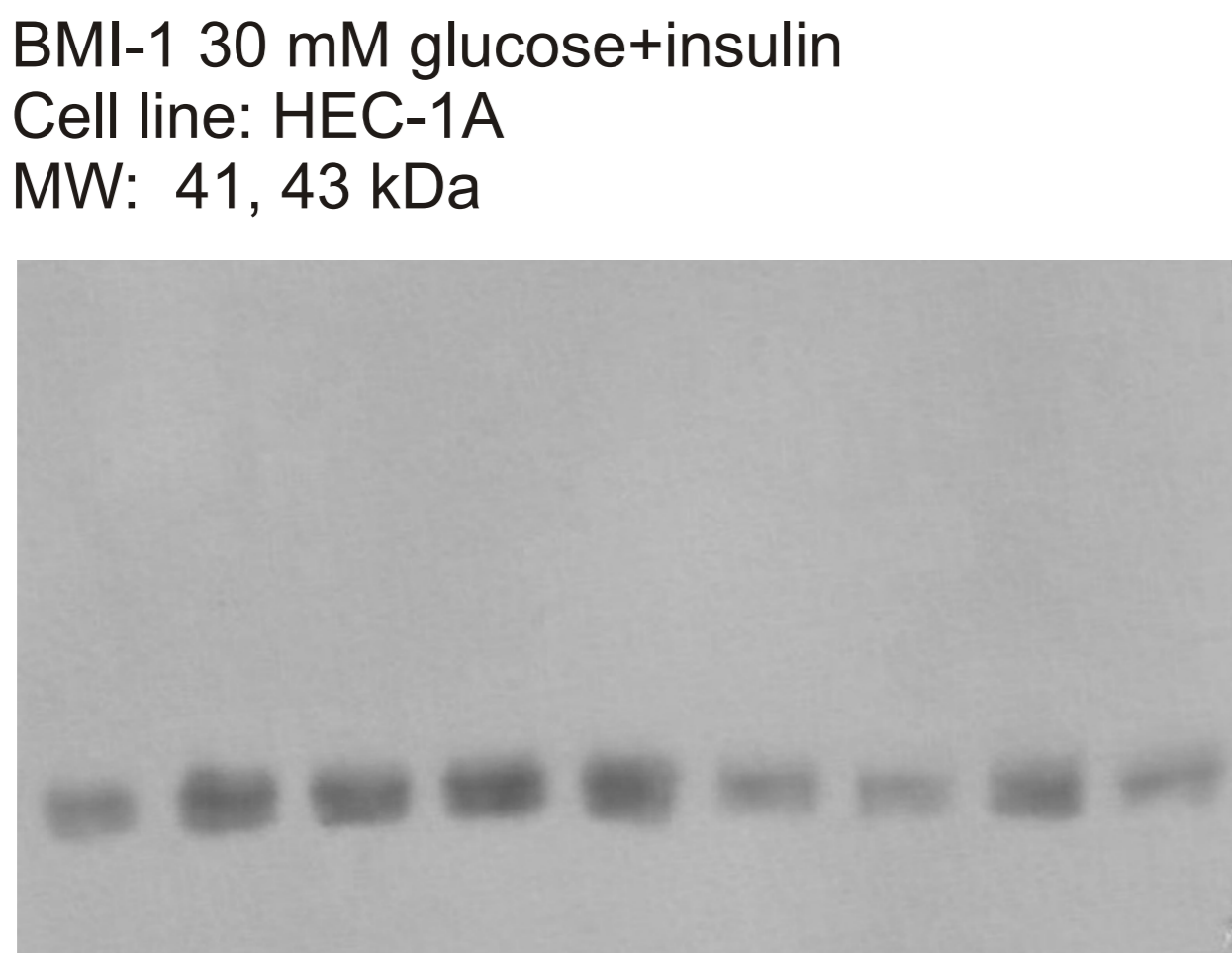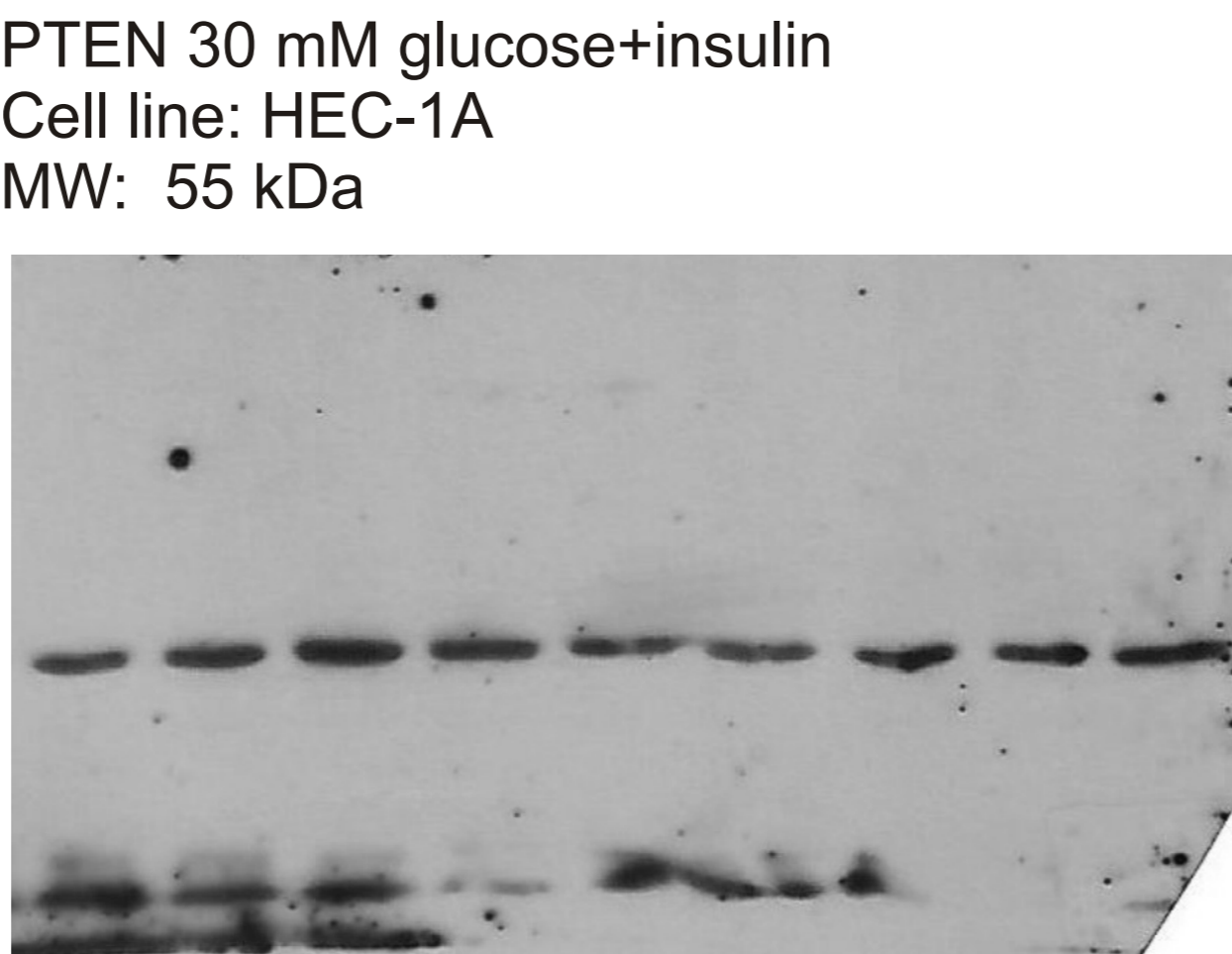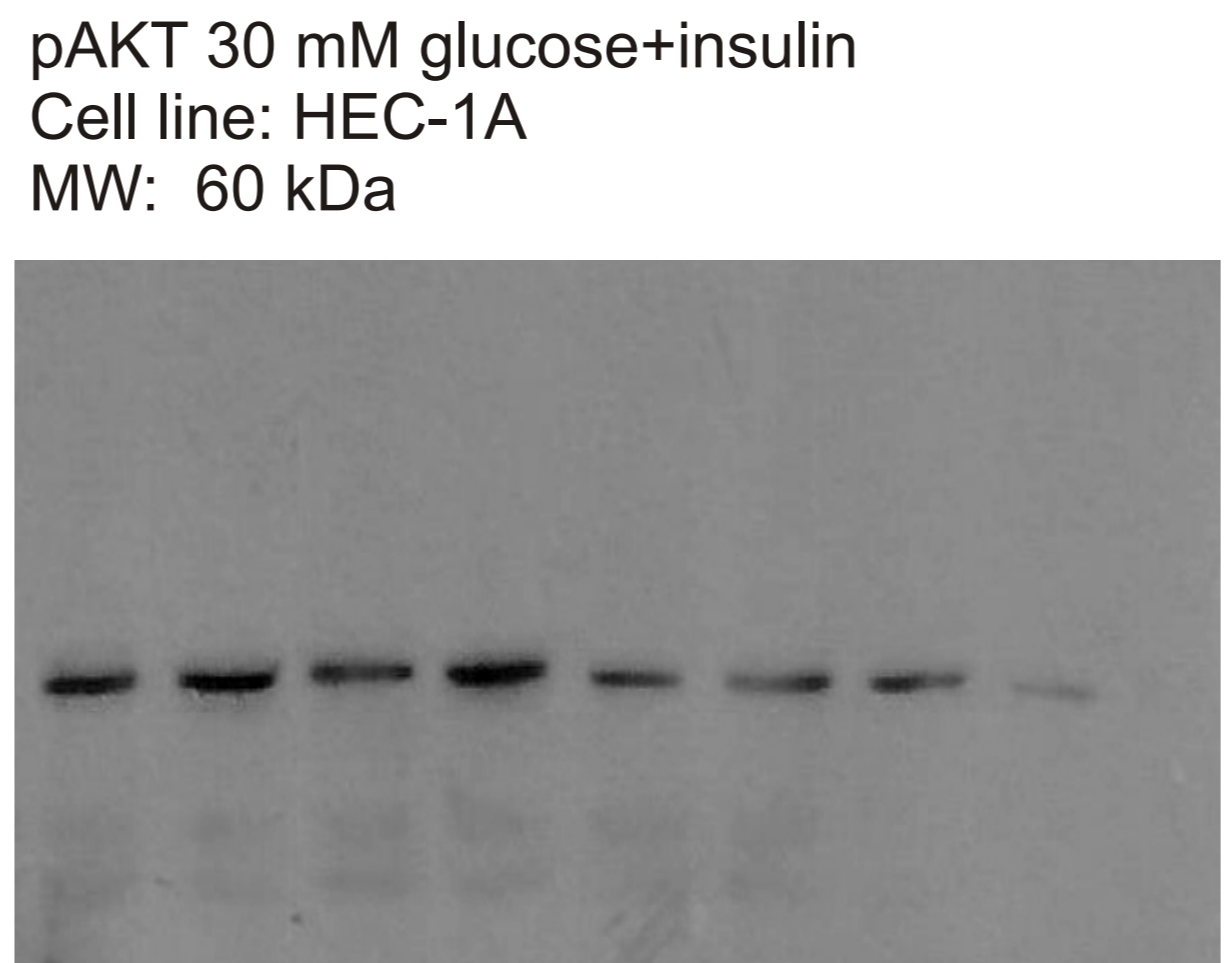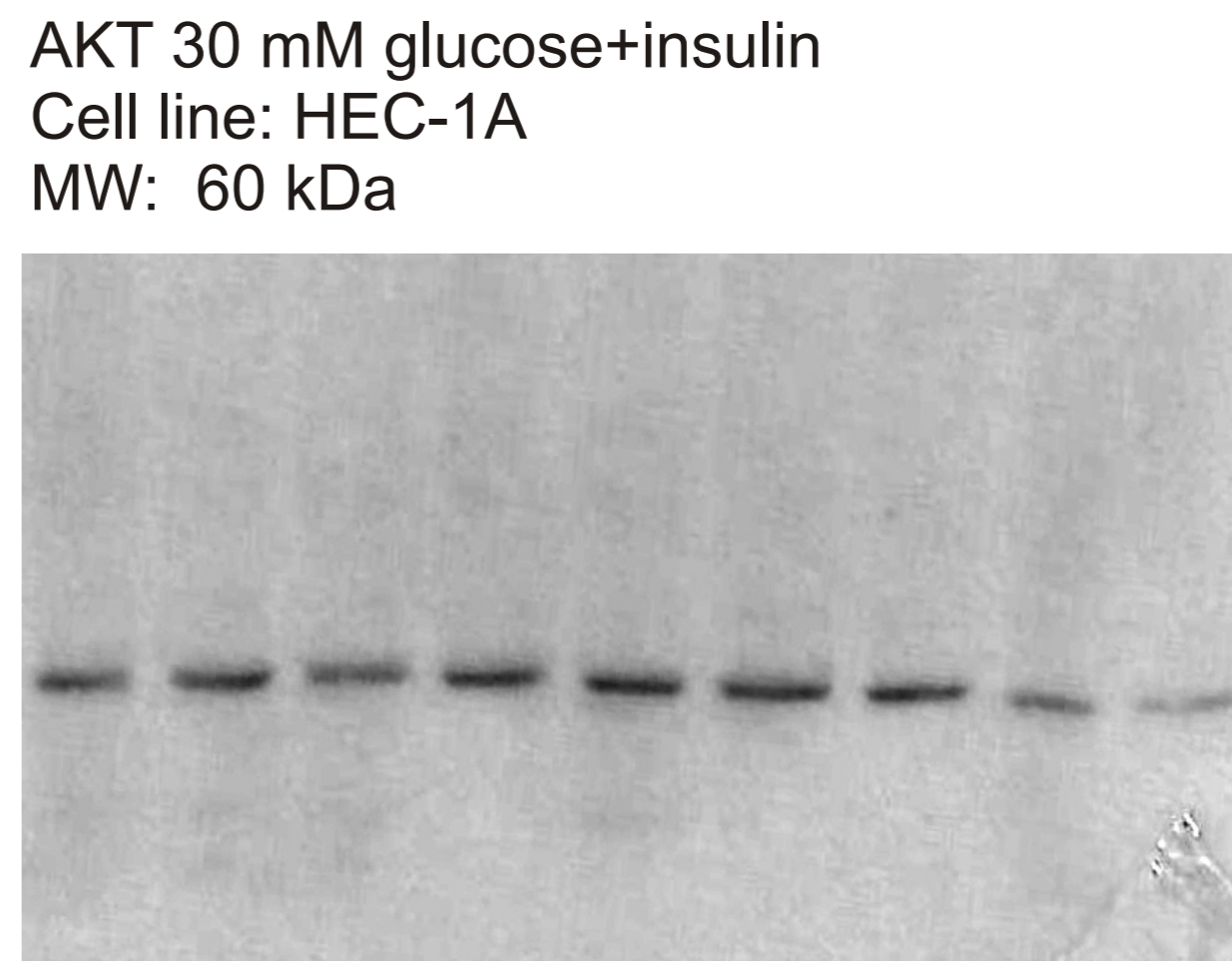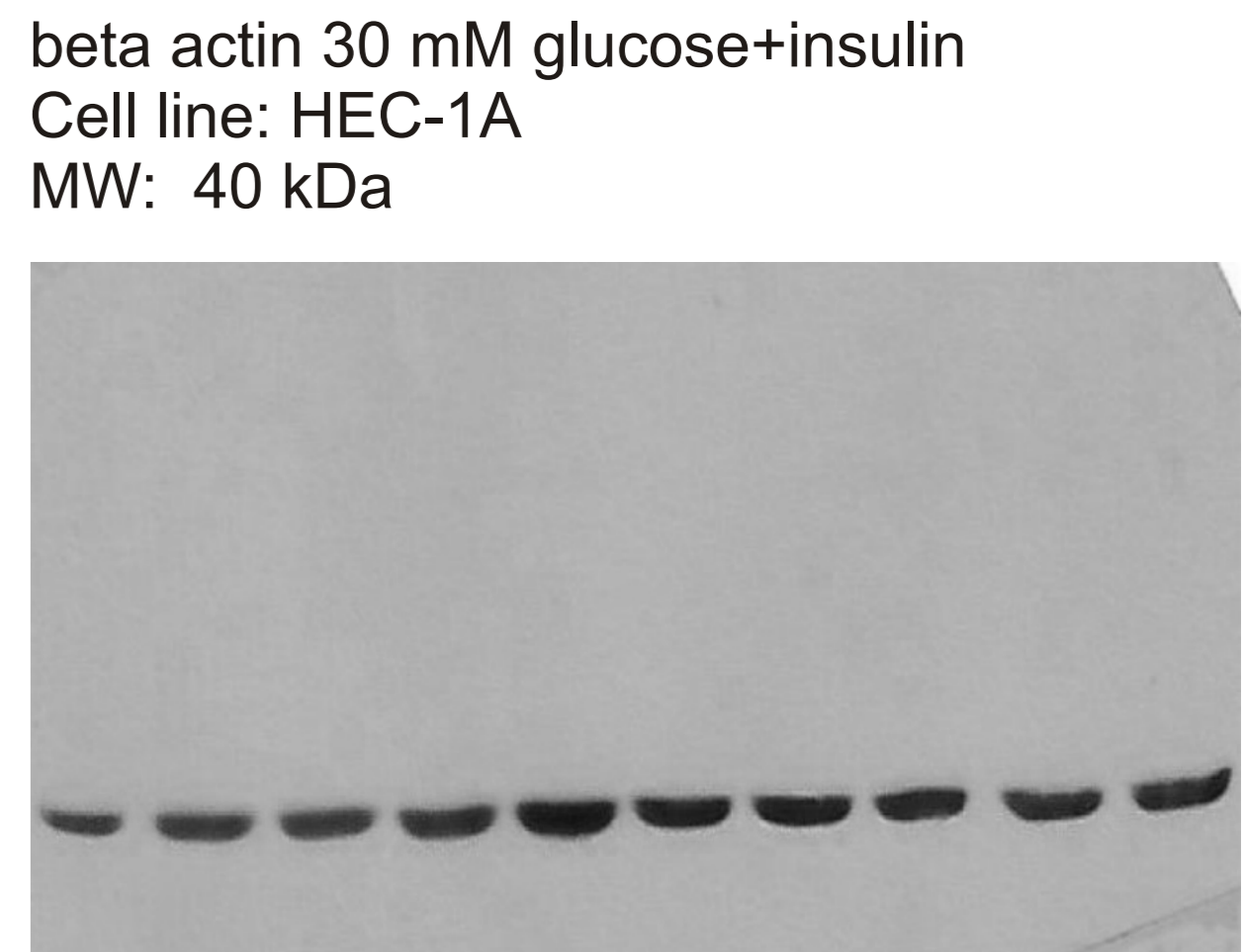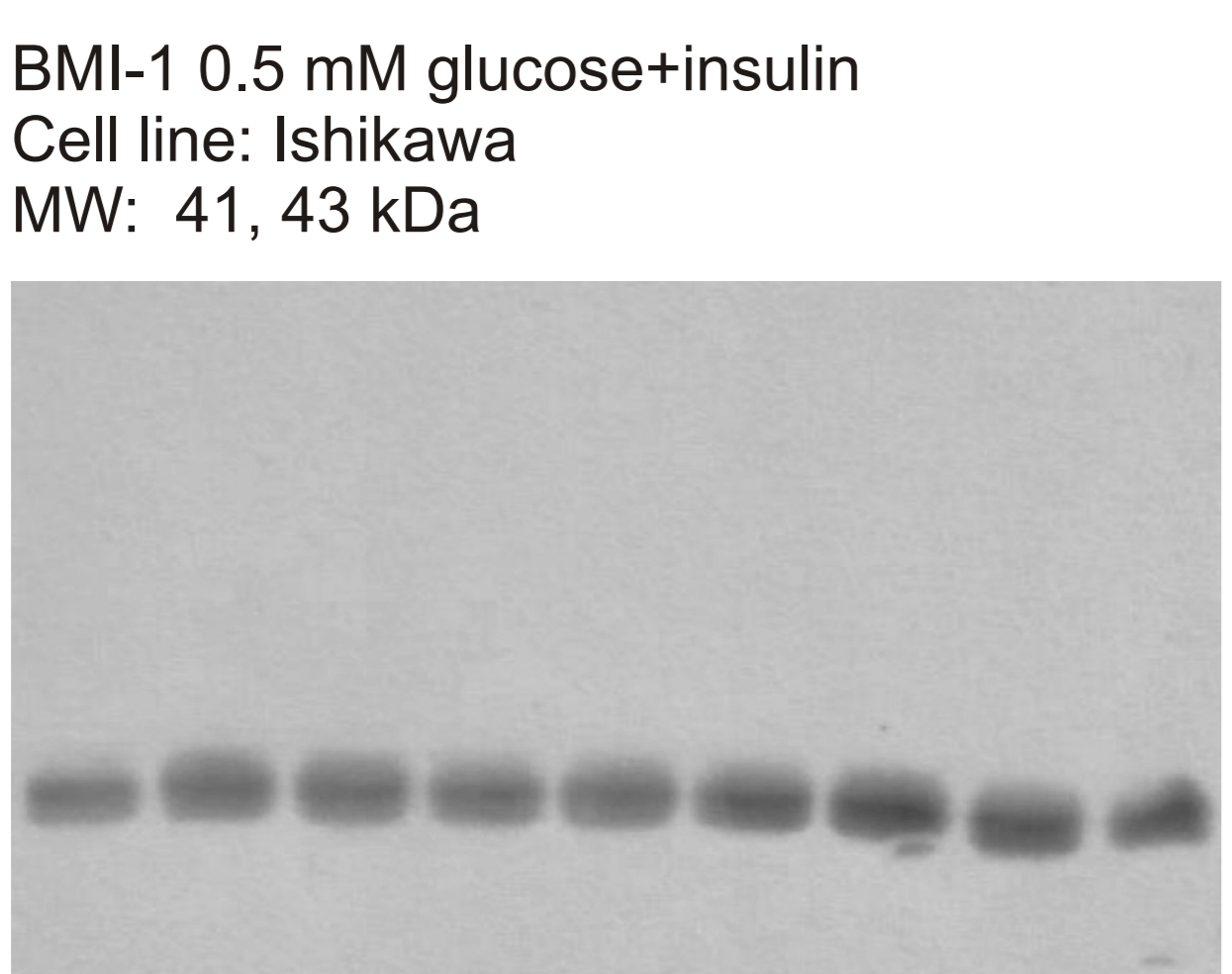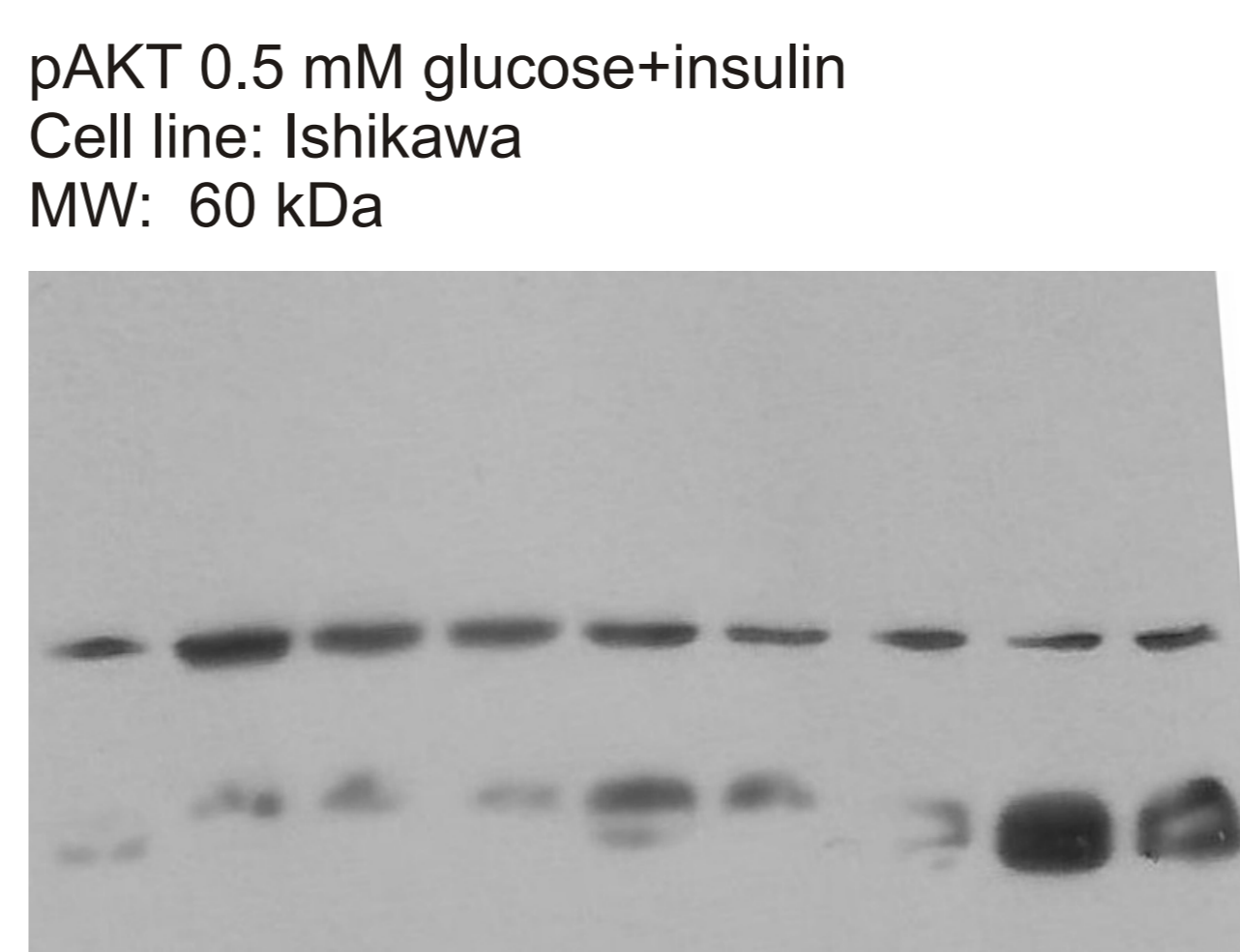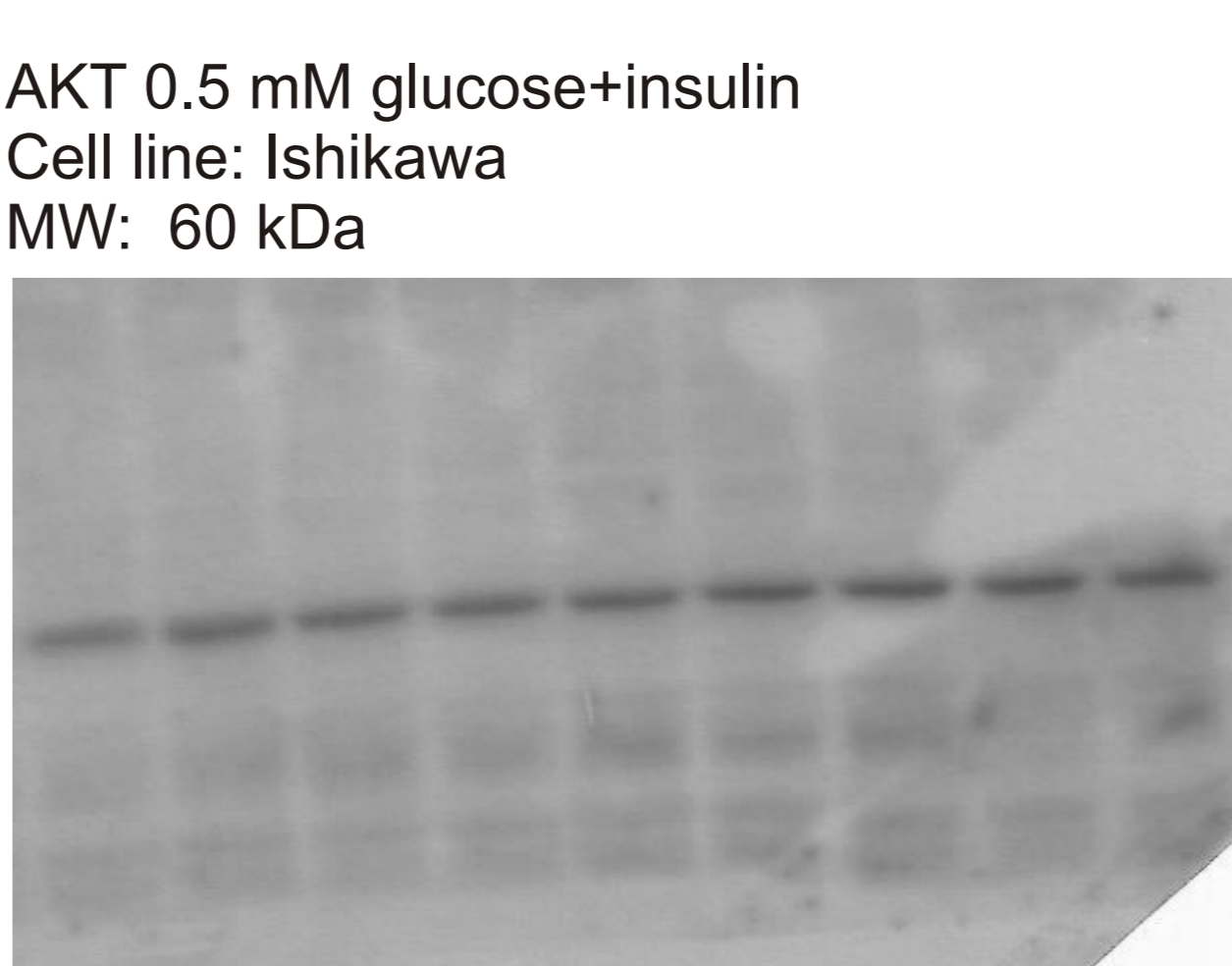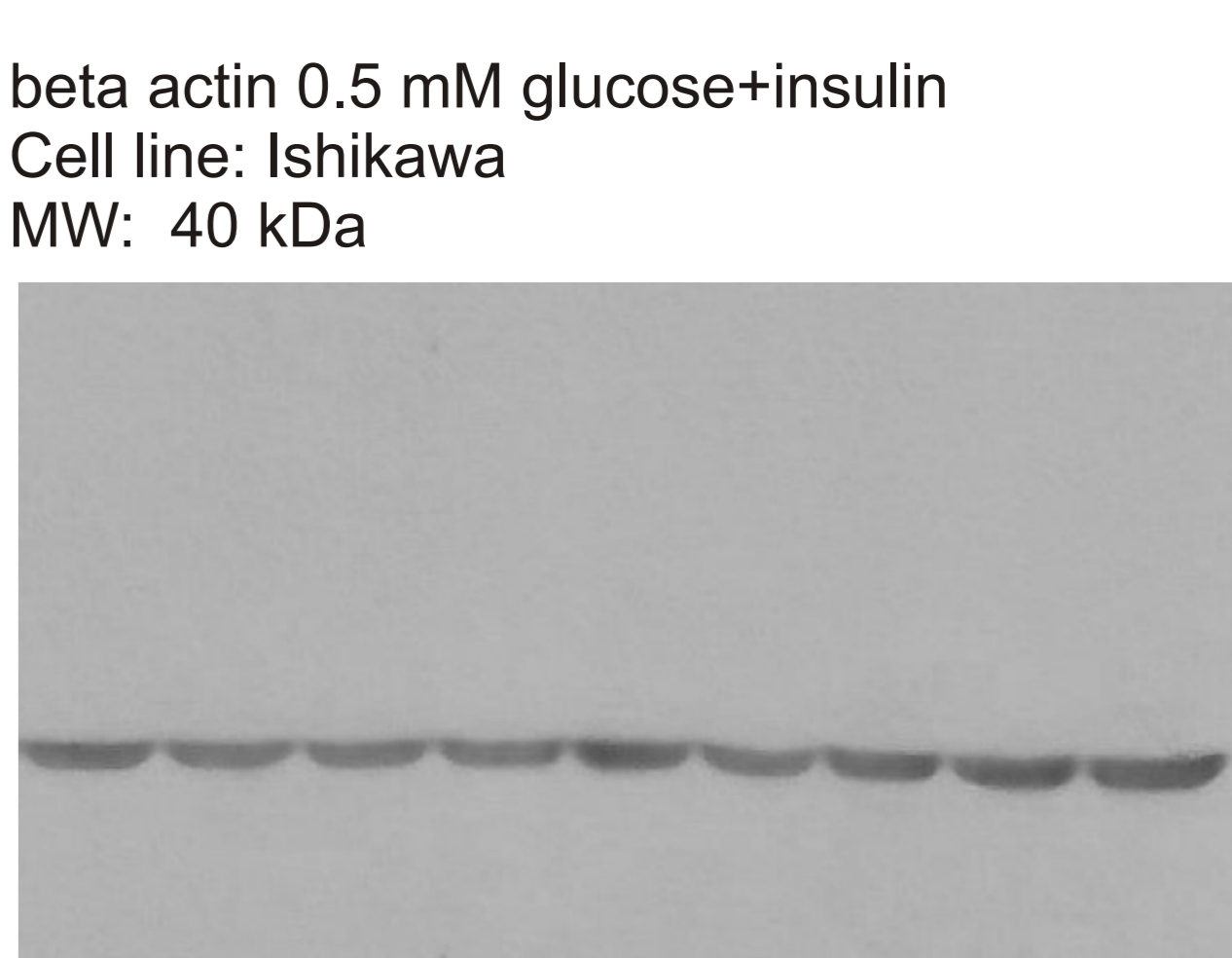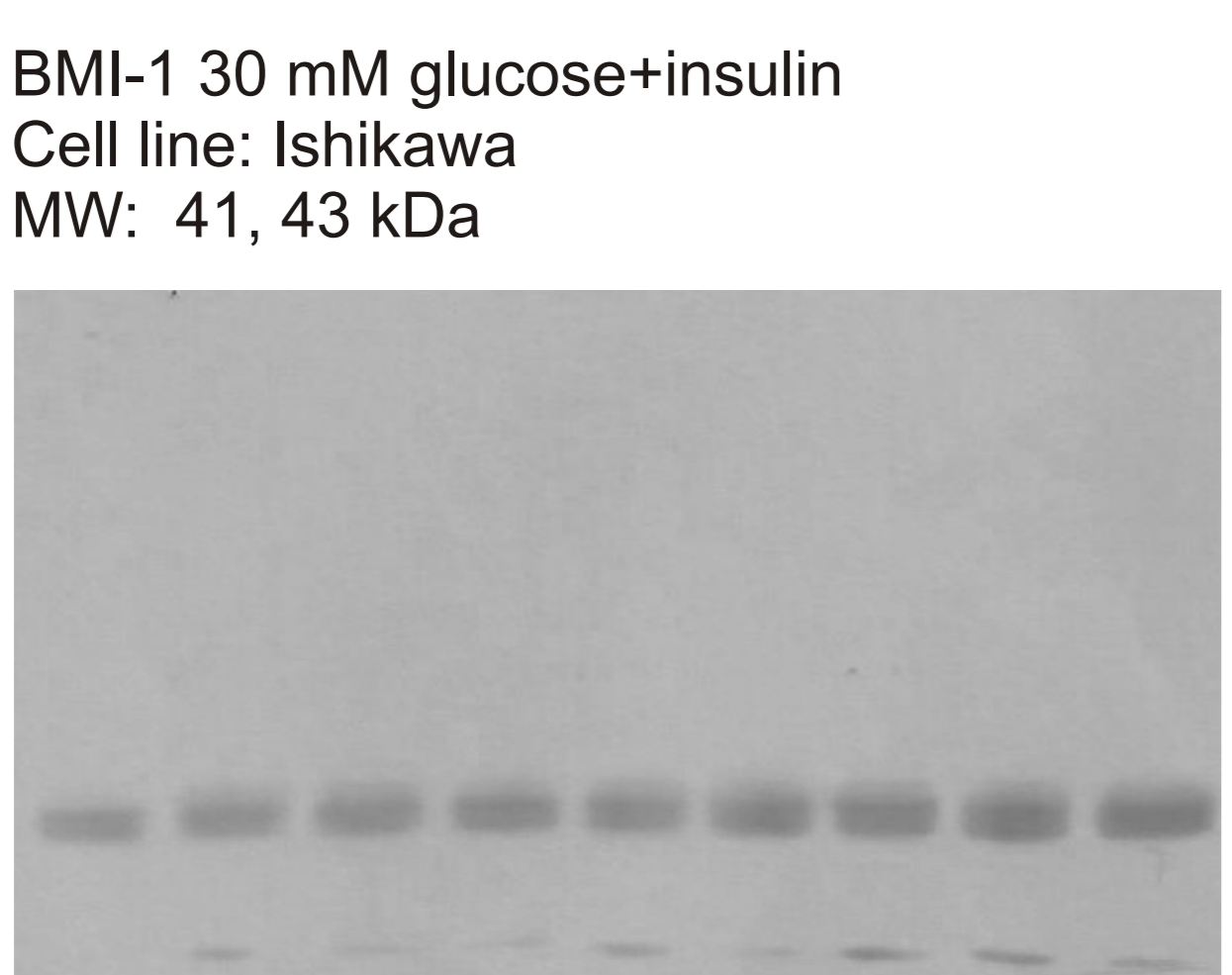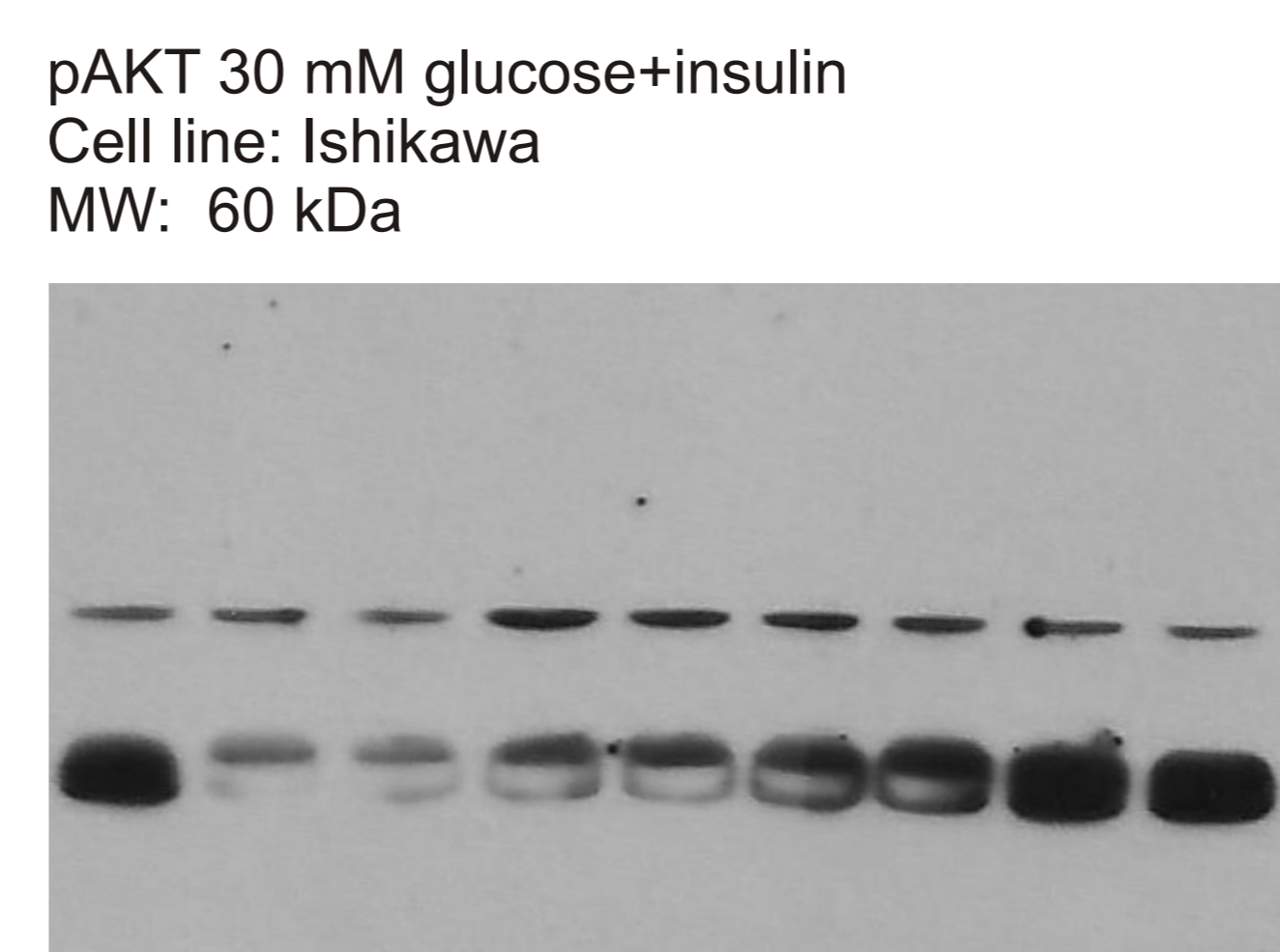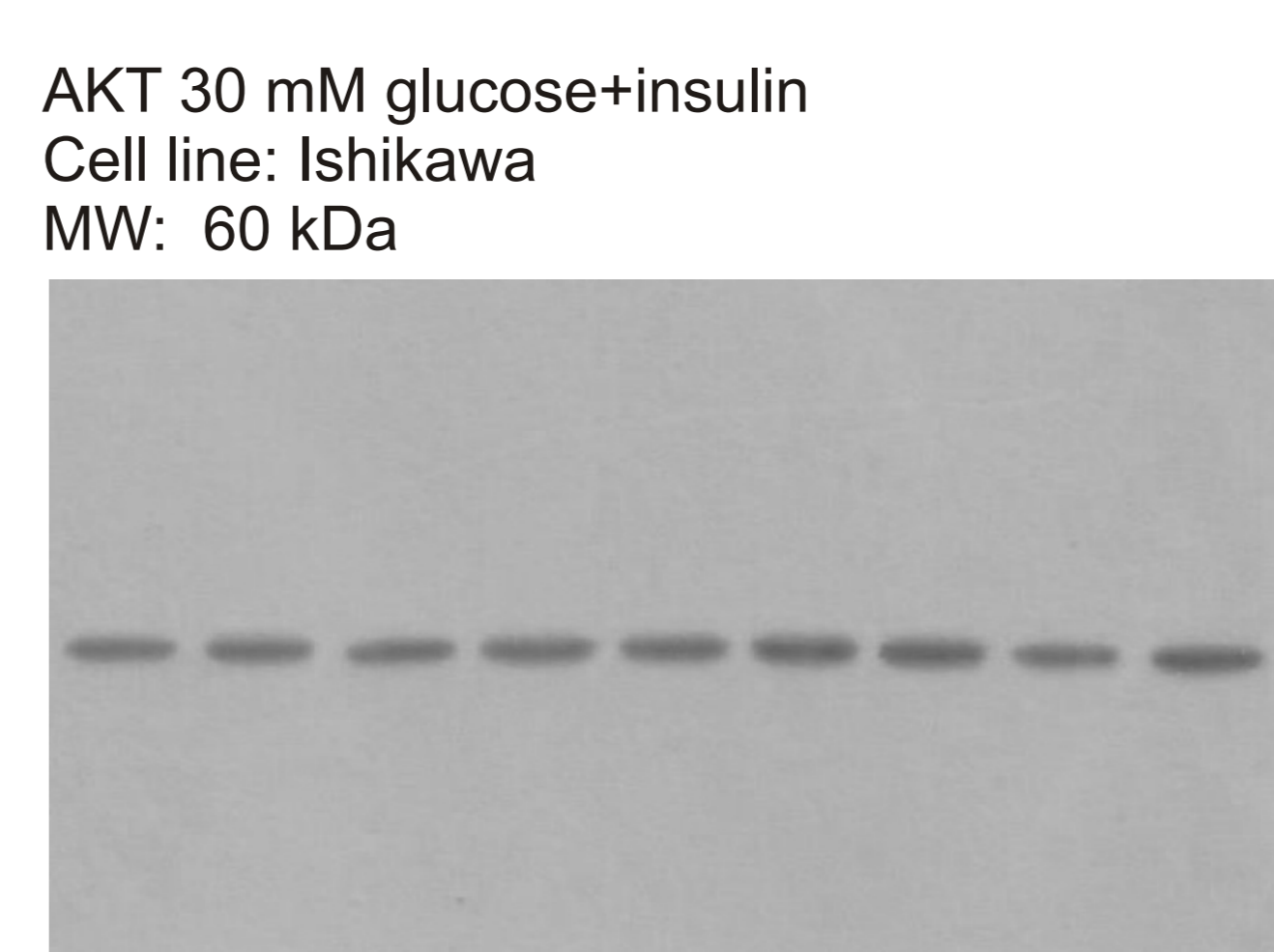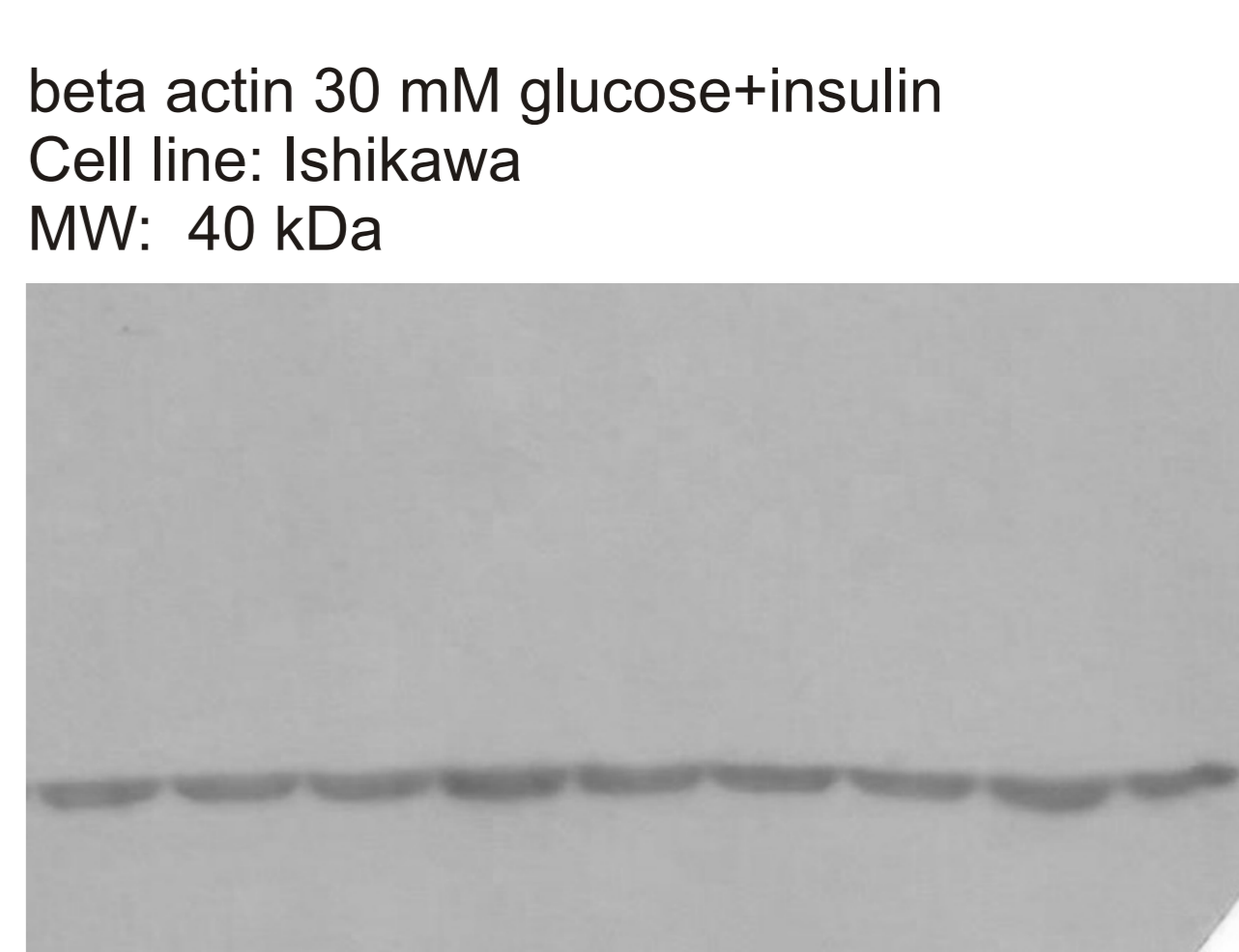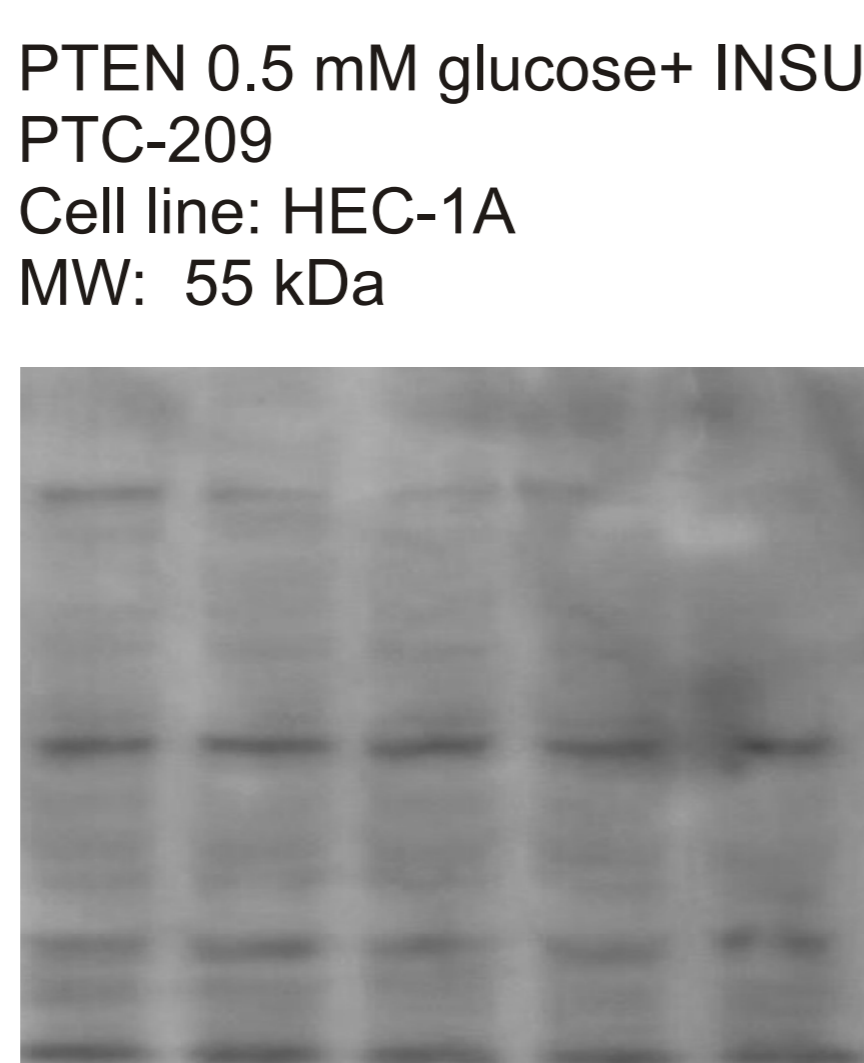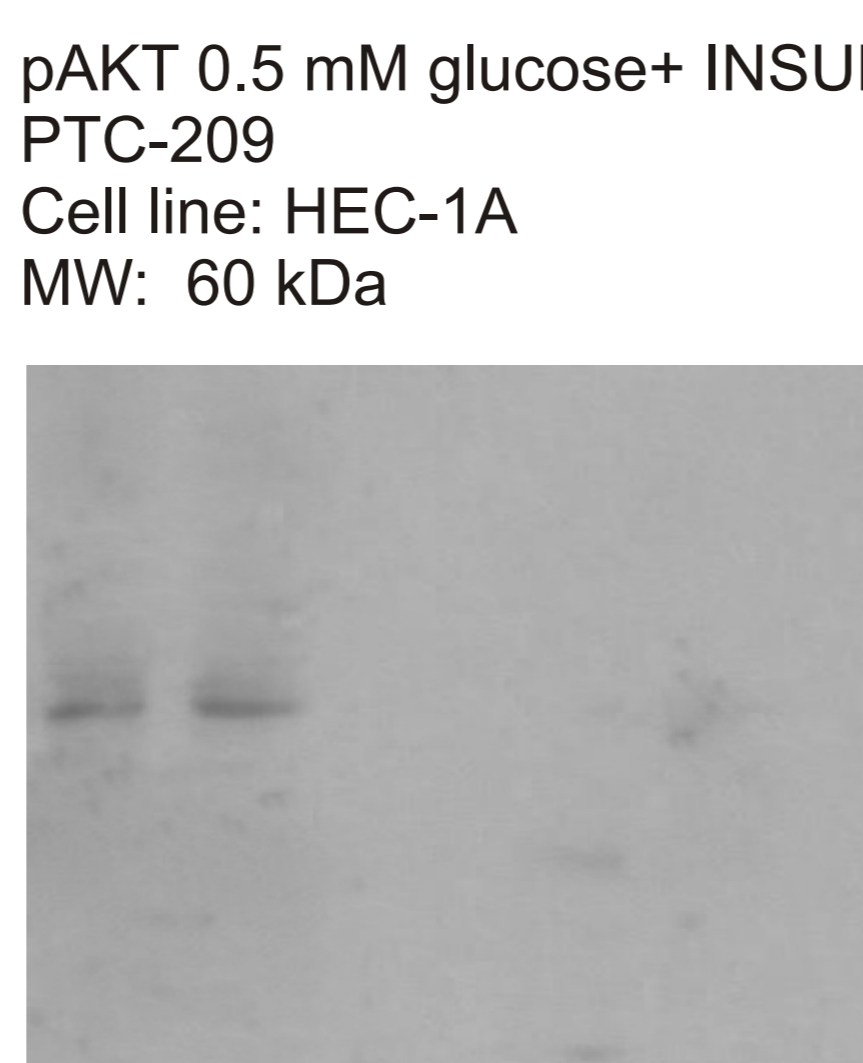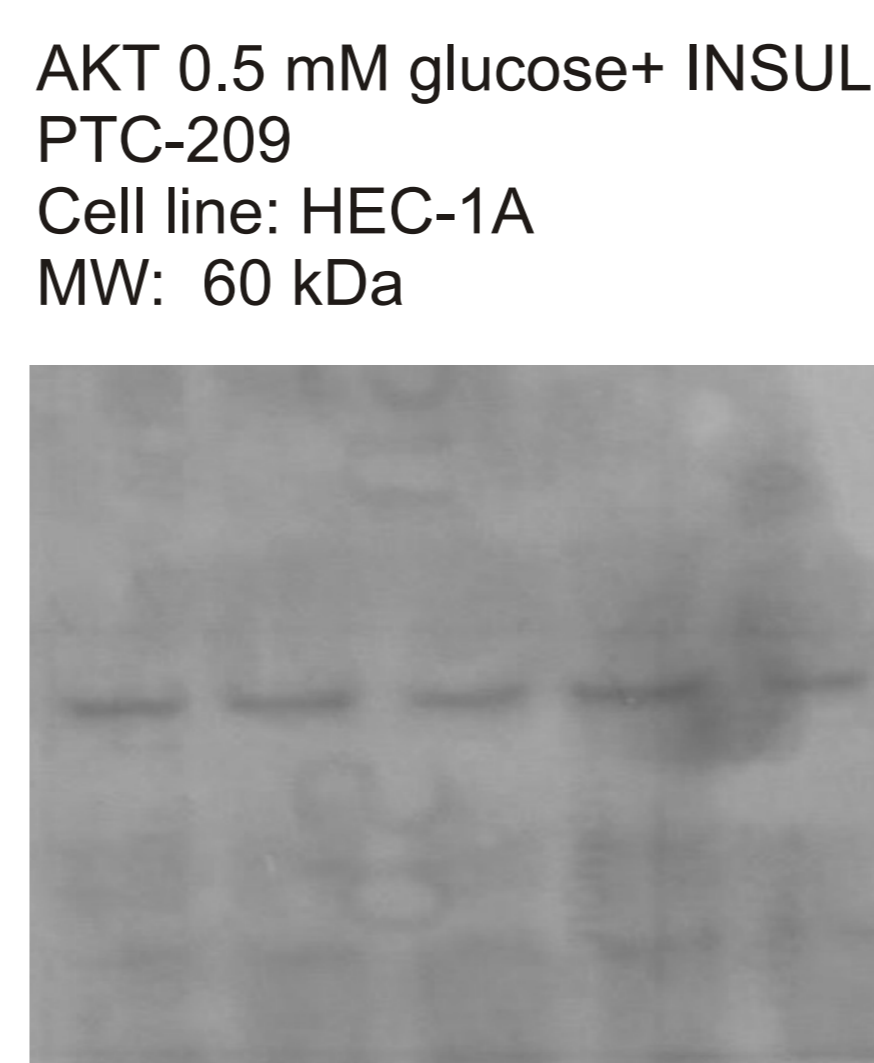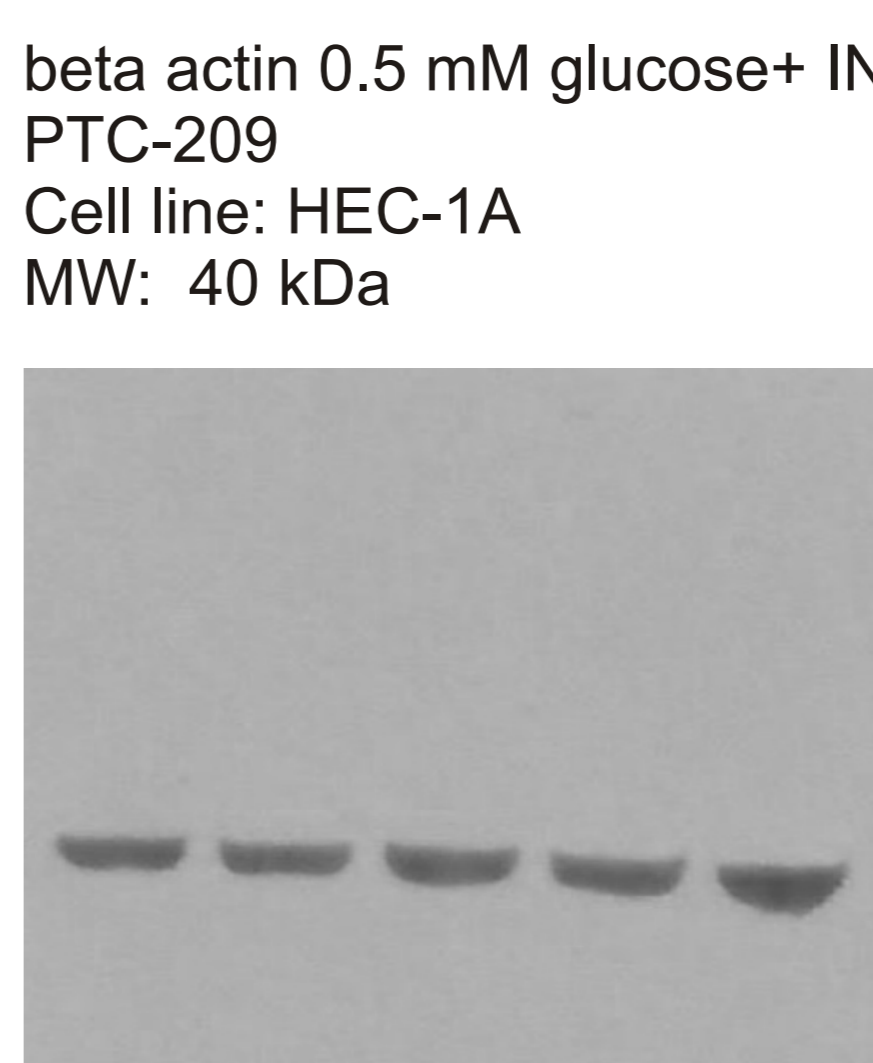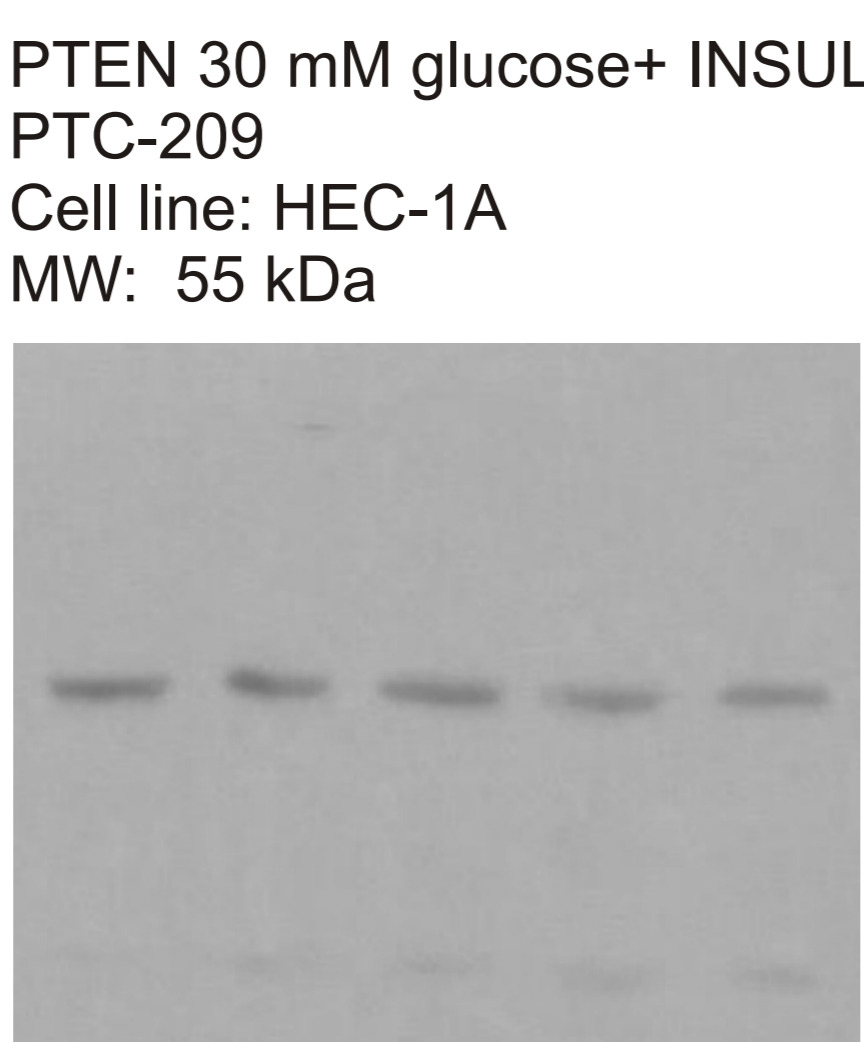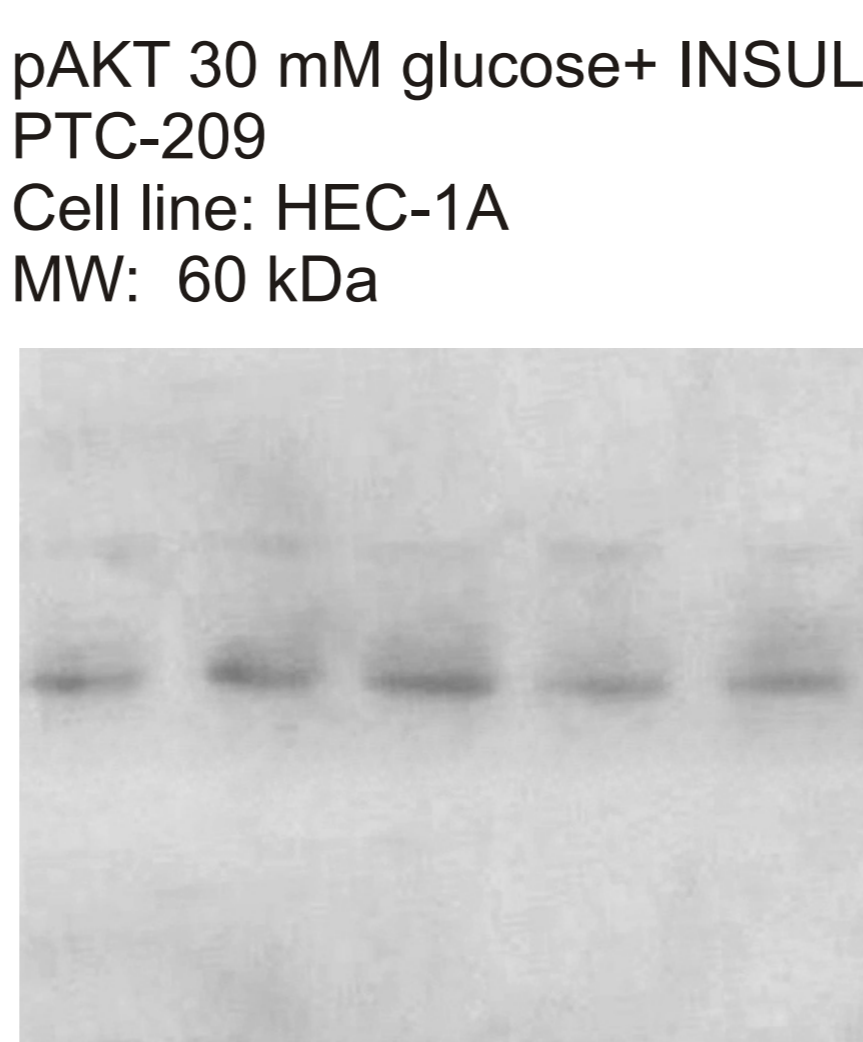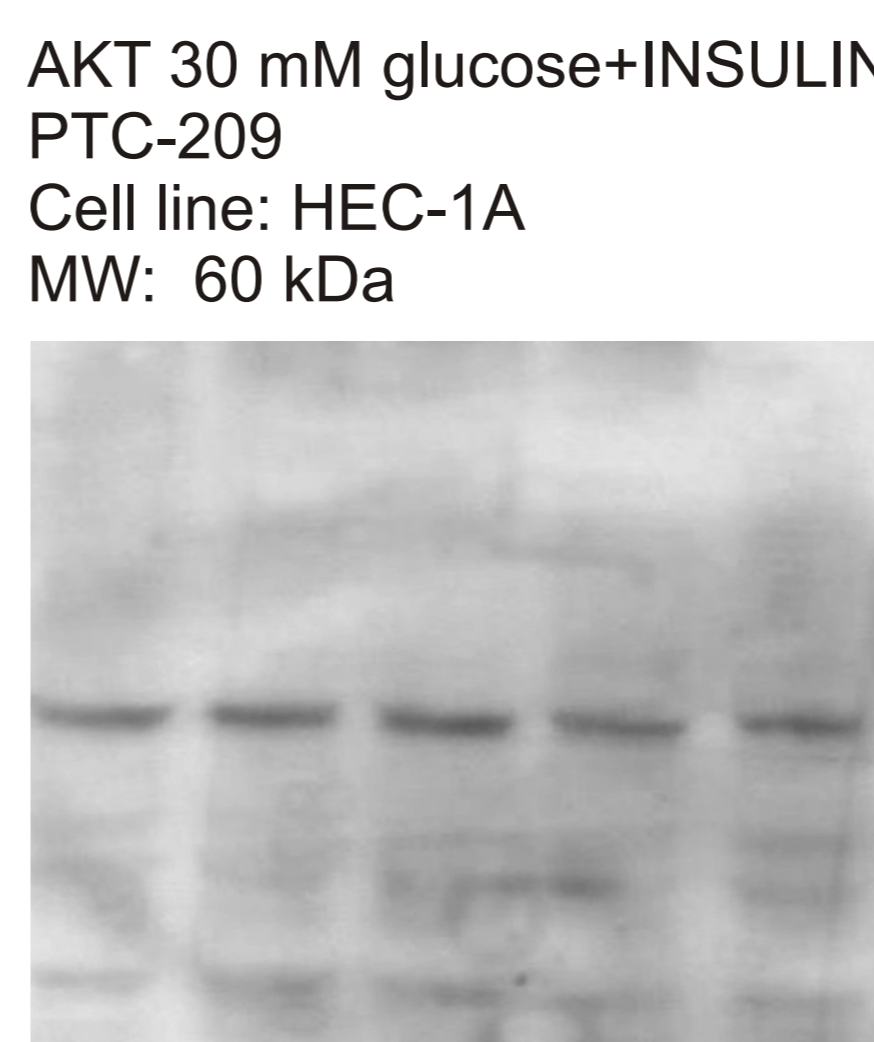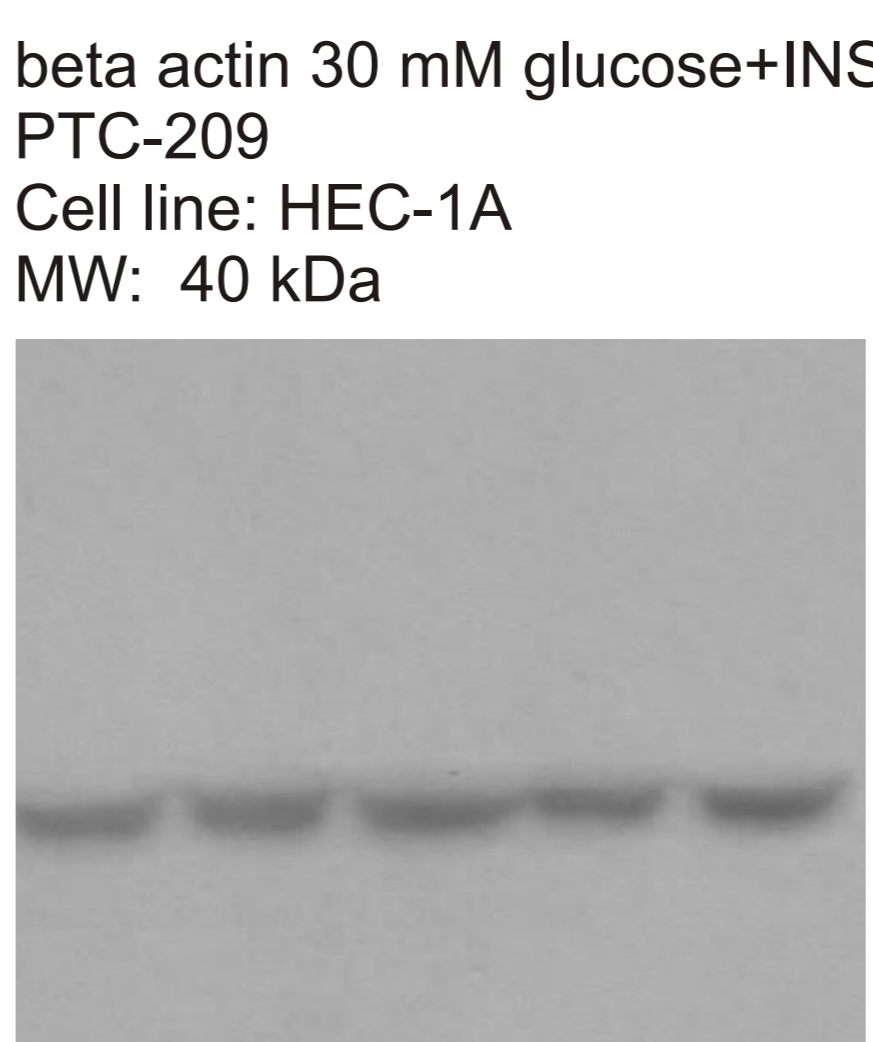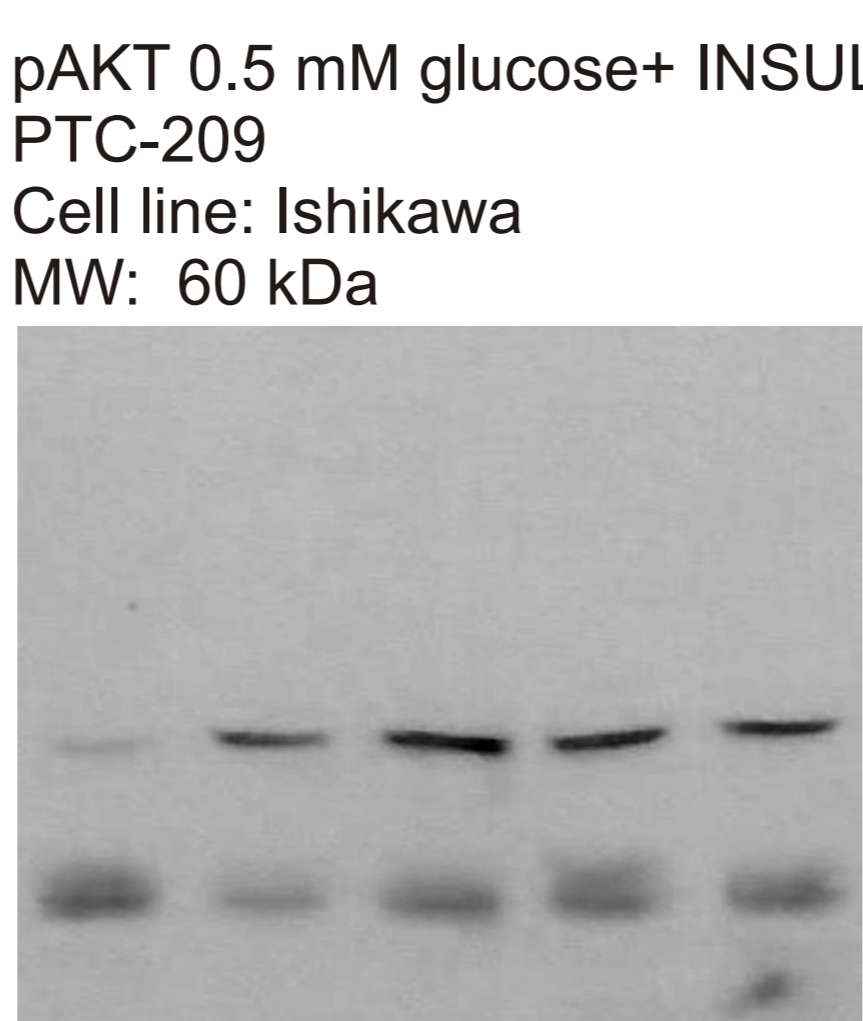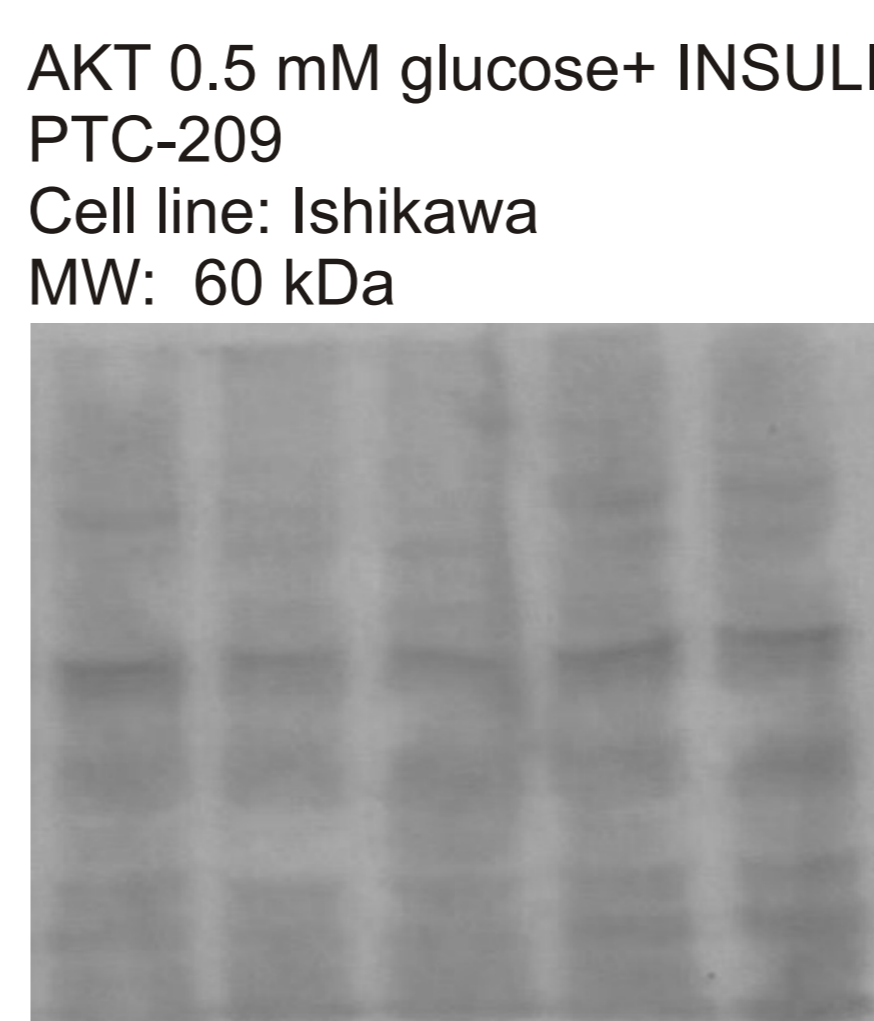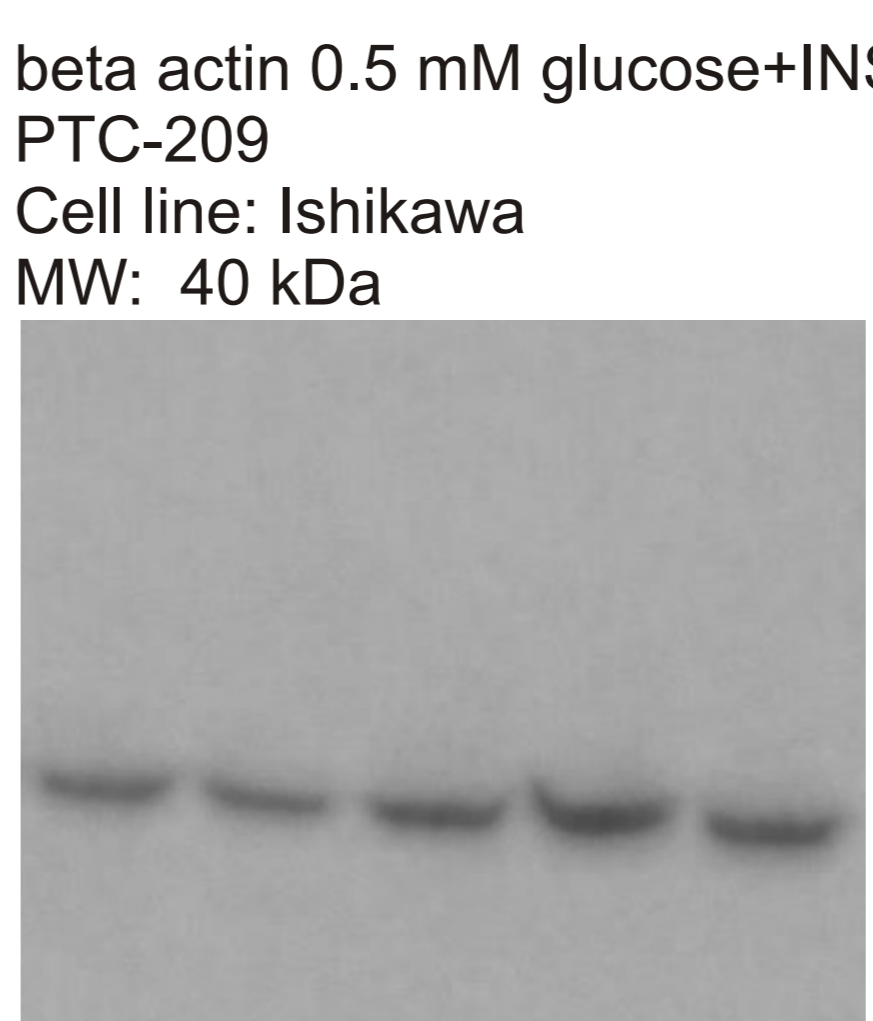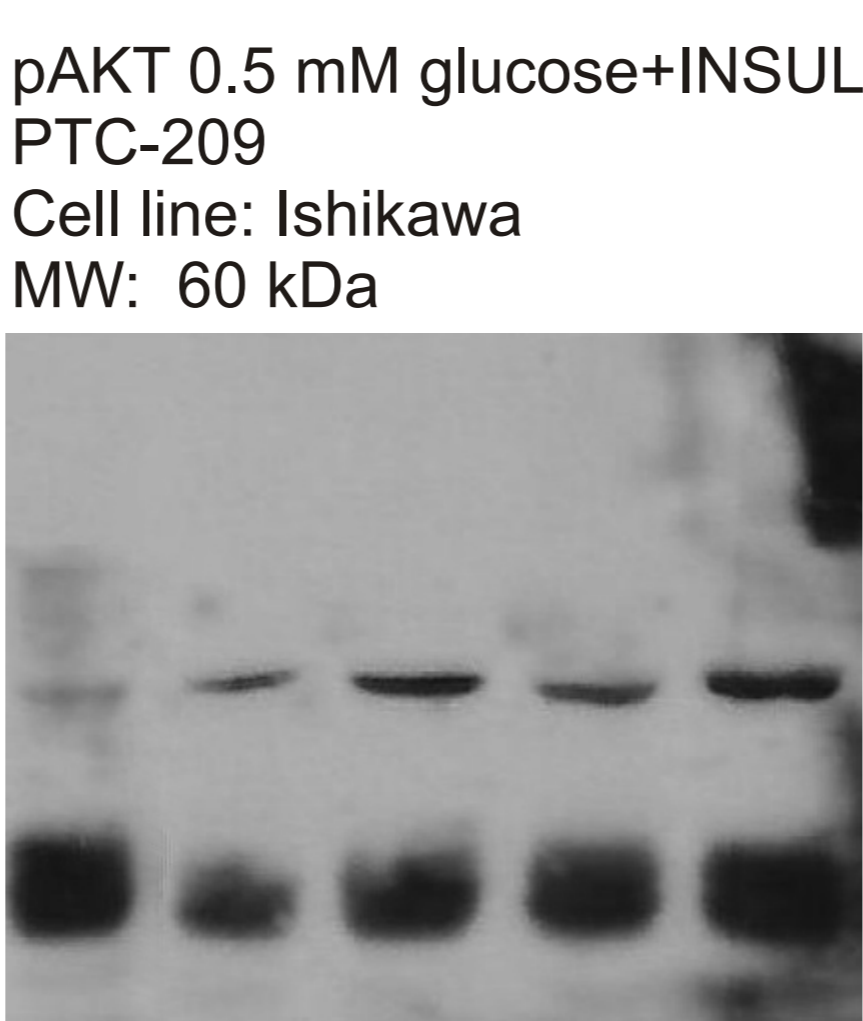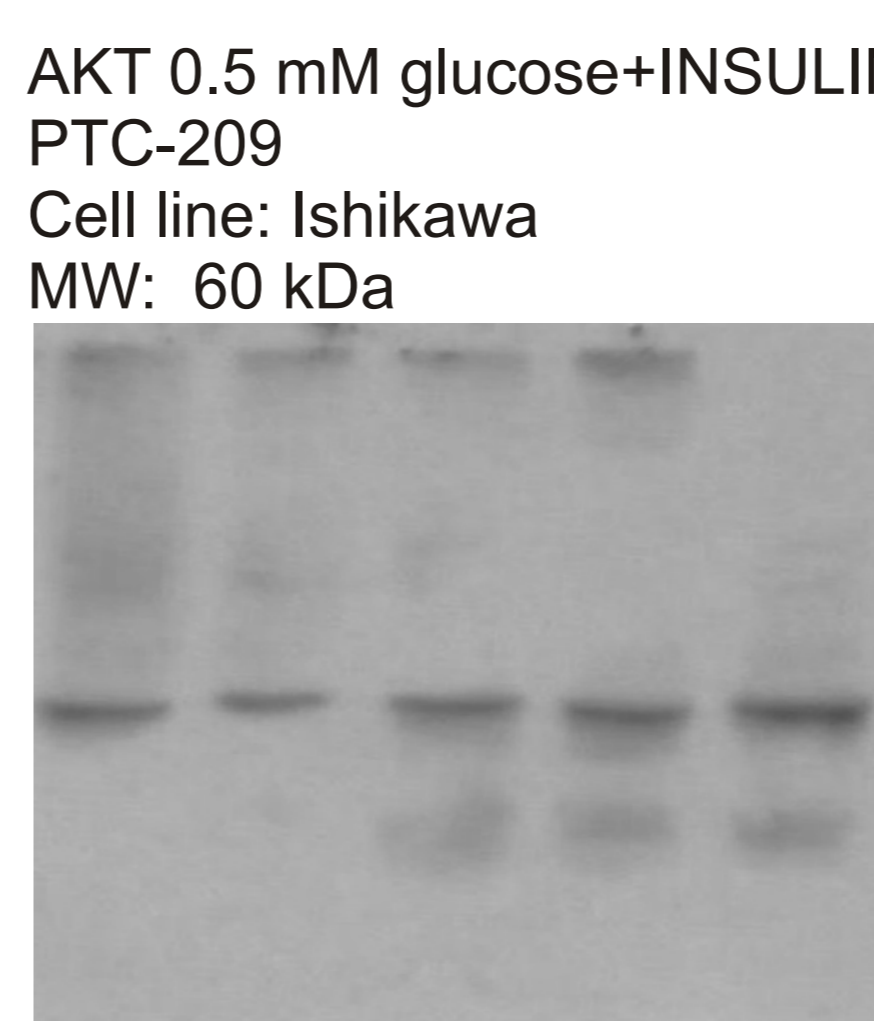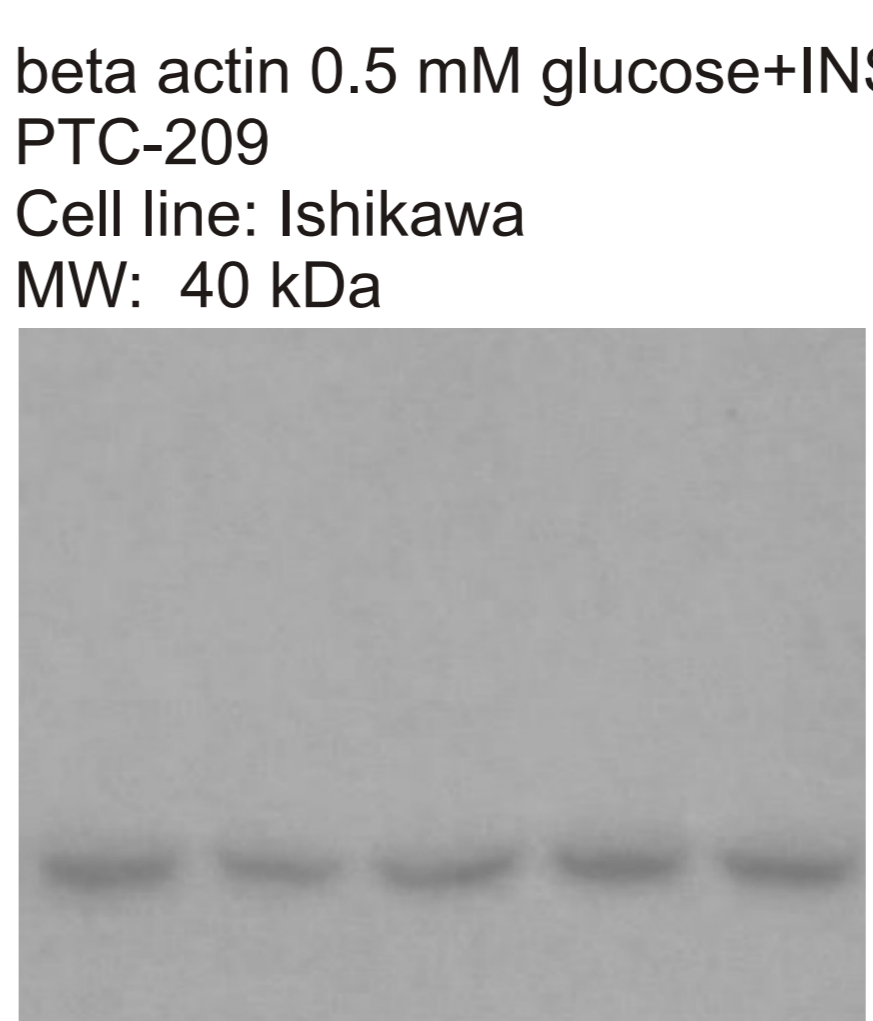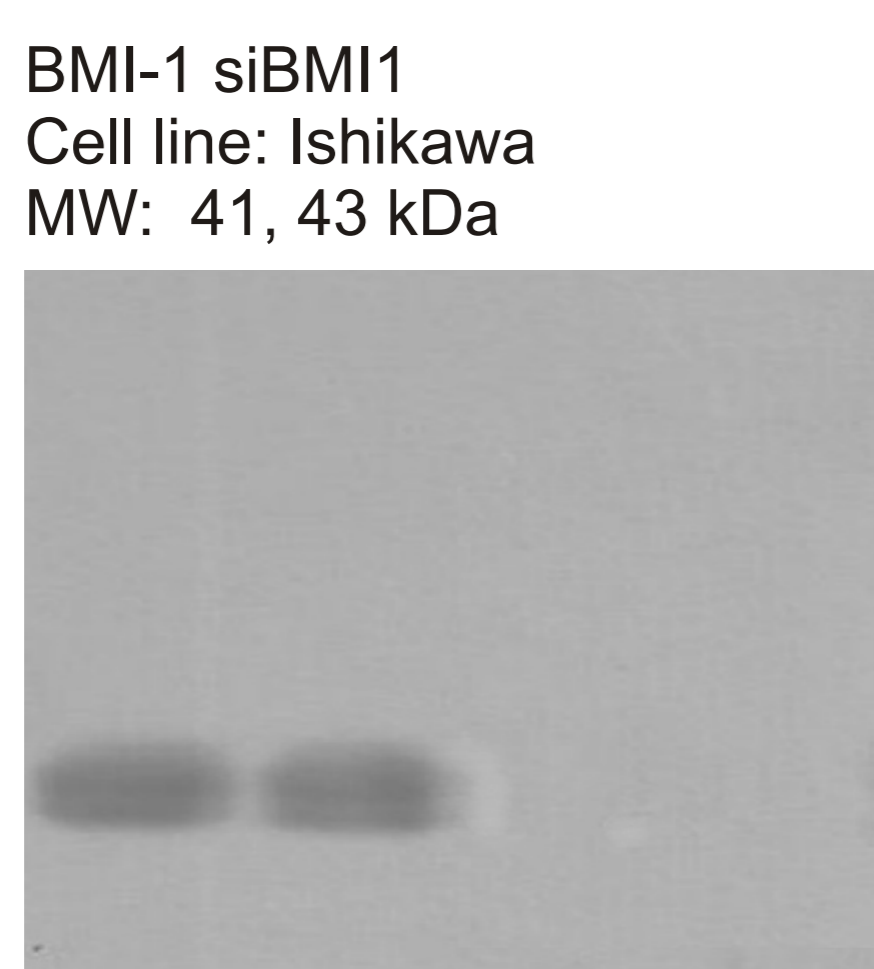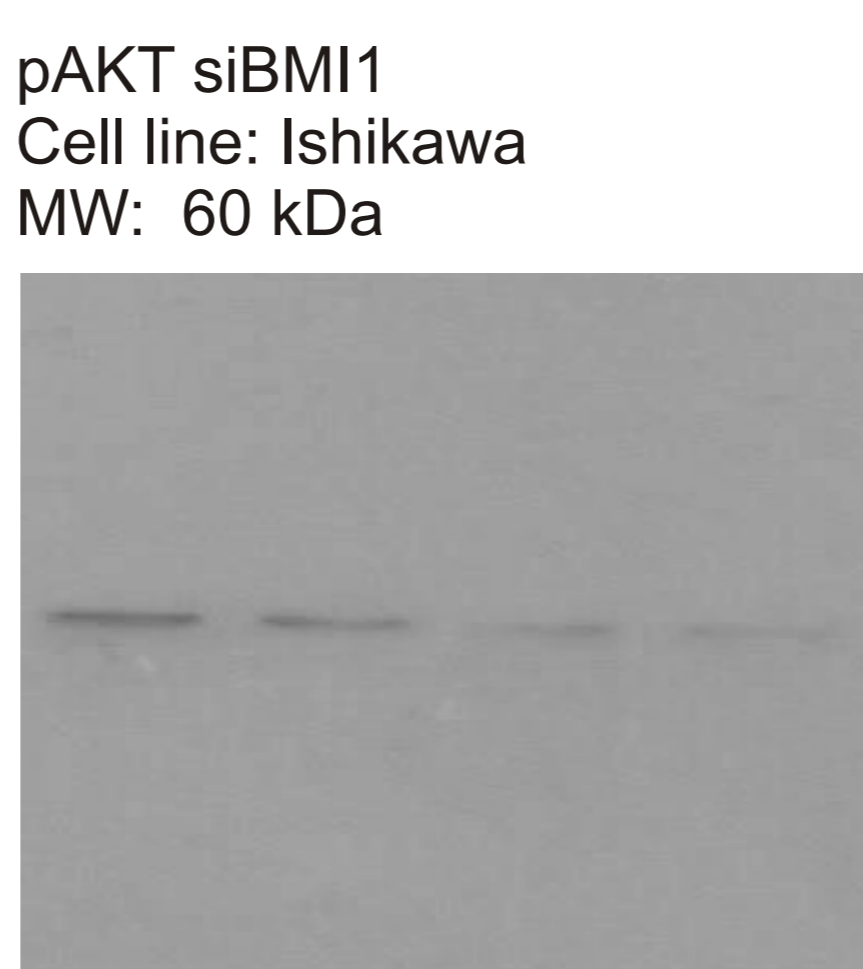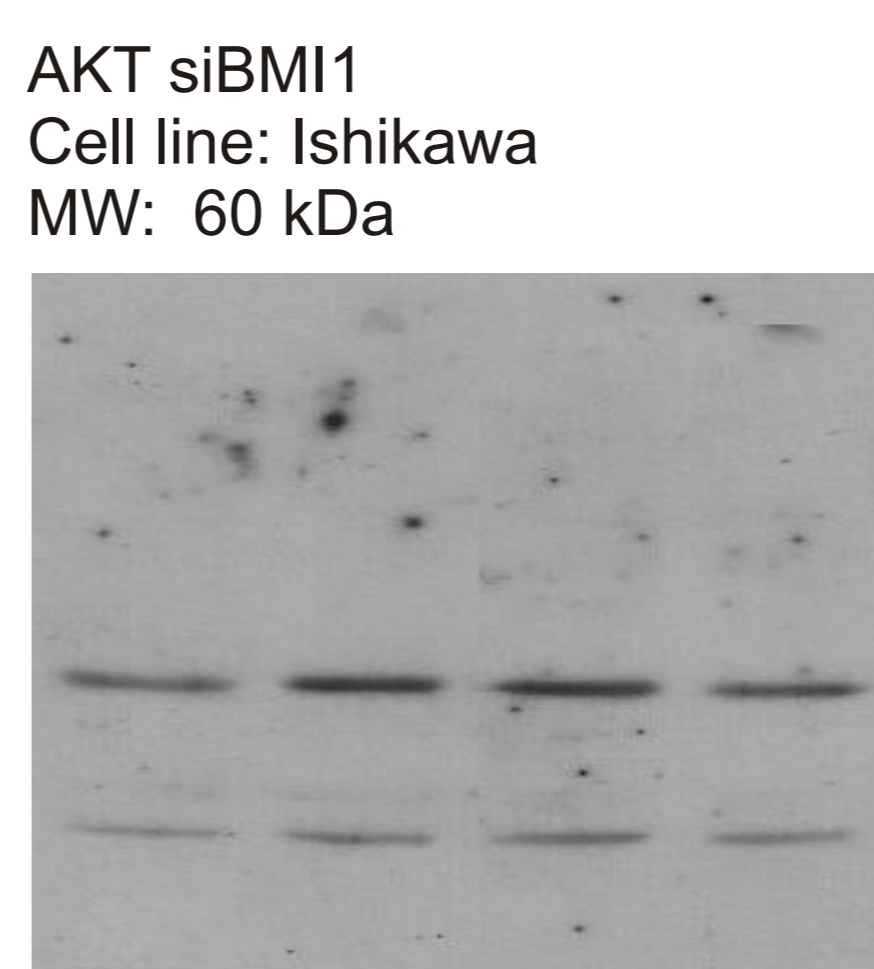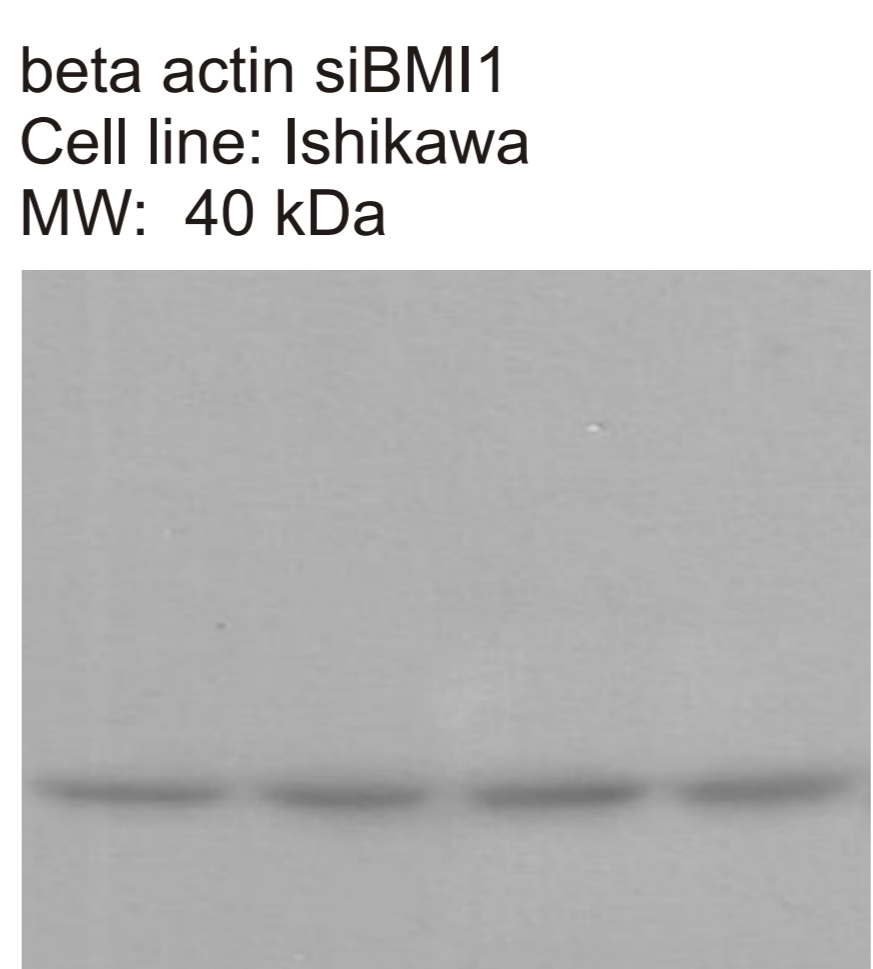

Supplement: Supplementary file 1 [file cancers-14-05947-s001.zip › cancers-1973946-File S1-wester blots.pdf]
